# Recent Advance in Synaptic Plasticity Modulation Techniques for Neuromorphic Applications

**DOI:** 10.1007/s40820-024-01445-x

**Published:** 2024-06-06

**Authors:** Yilin Sun, Huaipeng Wang, Dan Xie

**Affiliations:** 1 School of Integrated Circuits and Electronics, Beijing Institute of Technology, Beijing, 100081 People’s Republic of China; 2 School of Integrated Circuits, Beijing National Research Center for Information Science and Technology (BNRist), Tsinghua University, Beijing, 100084 People’s Republic of China

**Keywords:** Plasticity modulation, Dynamic plasticity, Chemical techniques, Programmable operation, Neuromorphic sensing

## Abstract

Manipulating the expression of synaptic plasticity of neuromorphic devices provides fascinating opportunities to develop hardware platforms for artificial intelligence. However, great efforts have been devoted to exploring biomimetic mechanisms of plasticity simulation in the last few years. Recent progress in various plasticity modulation techniques has pushed the research of synaptic electronics from static plasticity simulation to dynamic plasticity modulation, improving the accuracy of neuromorphic computing and providing strategies for implementing neuromorphic sensing functions. Herein, several fascinating strategies for synaptic plasticity modulation through chemical techniques, device structure design, and physical signal sensing are reviewed. For chemical techniques, the underlying mechanisms for the modification of functional materials were clarified and its effect on the expression of synaptic plasticity was also highlighted. Based on device structure design, the reconfigurable operation of neuromorphic devices was well demonstrated to achieve programmable neuromorphic functions. Besides, integrating the sensory units with neuromorphic processing circuits paved a new way to achieve human-like intelligent perception under the modulation of physical signals such as light, strain, and temperature. Finally, considering that the relevant technology is still in the basic exploration stage, some prospects or development suggestions are put forward to promote the development of neuromorphic devices.

## Highlights

- This review delves into the recent progress in high-performance and multifunctional neuromorphic devices for artificial intelligence applications from a novel perspective of plasticity modulation.
- It provides an in-depth discussion on the plasticity modulation strategies by chemical techniques, device structure design and physical signal modulation for advanced neuromorphic applications.
- It offers the prospects of exploring novel plasticity modulation mechanisms and techniques for scaled neural networks and examining their potentials in multimodal collaborative neuromorphic systems.

## Graphical abstract

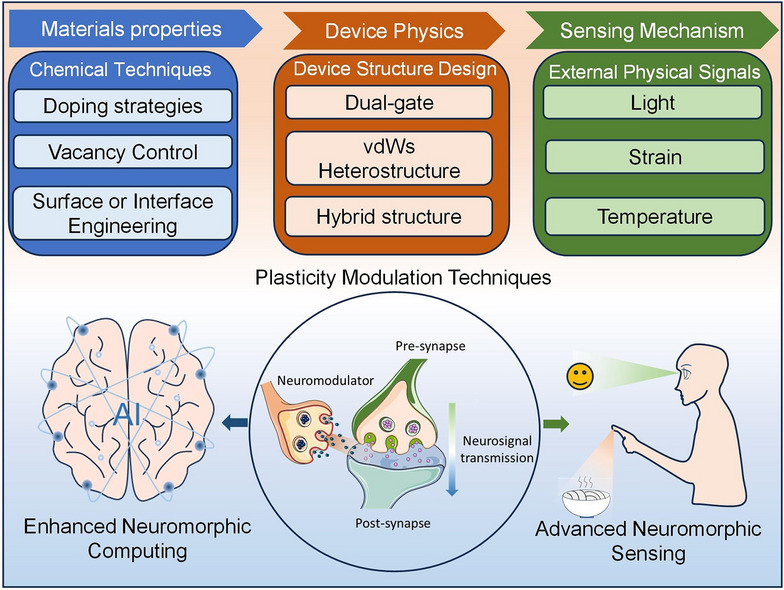

## Introduction

Synaptic electronics is a branch of electronics that aims to develop electrical systems that mimic the functions of biological neurons and synapses [1–3]. This interdisciplinary field combines cutting-edge research results from neuroscience, computer science, physical and material science, electrical engineering, and artificial intelligence. The ultimate goal of synaptic electronics is to create efficient and adaptive artificial neural systems that can learn and evolve, much like the human brain [4, 5]. In recent years, a variety of synapse-inspired hardware platforms have been developed to mimic the neuromorphic function of biological systems, which brings great insights into overcoming the limitations of conventional von Neumann architectures, for example, a mismatch between memory and CPU and the resulting latency and power consumption [6, 7]. However, artificial neural systems are still far inferior to biological ones in terms of scale and complexity in information processing due to the limited manufacturing technology, the lack of adaptive ability and the low level of plasticity. Even so, it is noted that the goal of artificial neural systems is not necessarily to replicate the whole complexity of the human brain but to learn from its principles to create efficient artificial hardware platforms able to solve complex tasks. From this view, exploring the approach to achieving high-level plasticity of synaptic devices is of great significance because synaptic plasticity is the foundation of learning, memory, and development in neural circuits[8].

Synaptic devices, based on the theory of synaptic electronics, utilize artificial electronic devices such as two-terminal memristors and three-terminal field-effect transistors (FETs) to simulate the plasticity of synapses in biomimetic organisms [9–11]. Specifically, memristors based on nanomaterials such as two-dimensional (2D) materials have been demonstrated to be key components for neuromorphic computing due to their simplified device structure, high-density arrays, and ability to emulate biological synaptic plasticity, providing possibilities for the development of artificial intelligence from a hardware perspective [12–14]. Before further discussions, two questions must be answered: (i) what is the synaptic plasticity? (ii) how can it be emulated in artificial devices? Firstly, the plasticity of biological synapses represents the experience-dependent change in connectivity between neurons, which can be emulated by the continuous, repeatable, and non-volatile change of device electrical parameters (such as current or conductivity), also known as synaptic weight, under excitation signals. According to the time scale of synaptic weight change, plasticity can be classified into short-term plasticity (STP) which lasts for several seconds or minutes, and long-term plasticity (LTP) which indicates a permanent change of synaptic weight [15–17]. Moreover, the transition from STP to LTP can be achieved by repeated stimuli [18]. Besides, artificial synaptic devices are also endowed with other synaptic functions such as pair-pulse facilitation (PPF), one of typical STP, spike-timing-dependent plasticity (STDP) and excitatory and inhibitory plasticity that refers to the enhanced or weakened synaptic weight under stimuli, respectively [19]. Secondly, the physical mechanism behind synaptic plasticity emulation determines how synaptic function can be achieved in artificial devices. Biological synapse can simultaneously process and store information in an in-memory computing manner, which is completely different from von Neumann architecture. Following this principle, current artificial synaptic devices have evolved from structures or devices with storage and memory functions, including sandwich structure memristors [20], floating-gate structure [21, 22], electrolyte-gated transistor [23] and ferroelectric-gate transistors [24, 25]. The weights in artificial synaptic devices can undergo continuous changes and exhibit multiple conductance states under external stimulation, and the change of synaptic weight is associated with the temporal characteristics (frequency, period, spike duration and time sequence et al.) of applied pre-synaptic stimuli, which separates it from the conventional memory devices. Although great efforts have been devoted to designing and fabricating synaptic devices to emulate the plasticity of biological ones, the dynamics of such devices, however, have been not well achieved, which is essential for the complicated neuromorphic functions of living creatures [26].

The dynamics of synaptic plasticity refer to a controllable expression of synaptic functions in both the changing external environment and self-regulating internal environment of living organisms [27]. A similar idea can be also implemented in artificial synaptic devices to modulate the plasticity through a specific technique, which contributes to a better understanding of the working principles of artificial neural networks and inspires innovation in neuromorphic computation applications. However, most reported synaptic devices can only exhibit the ability to emulate static plasticity without effective modulation methods to realize dynamic plasticity. Table 1 summarizes the recent great efforts on plasticity modulation techniques [28–41]. The targets of synaptic plasticity modulation can be classified into two aspects: enhanced neuromorphic computing and advanced neuromorphic sensing. Neuromorphic computing depends on the synaptic weight updating rules and programmable or reconfigurable synaptic functions realized in neuromorphic devices, which means that optimization is needed for typical synaptic plasticity such as STP and LTP, potential and depression, and nonlinearity (NL) and symmetry. For example, the smaller value of NL has been demonstrated to contribute to a higher accuracy of artificial neural networks for pattern recognition [42]. To achieve more efficient neuromorphic computing, plasticity modulation techniques are needed to control the expression of synaptic plasticity and optimize the performance of synaptic devices. From Table 1, it can be inferred that the performance of synaptic devices is directly determined by the active material properties tuned by chemical techniques or the controllable carrier transport characteristics by specifically designed device structure. For neuromorphic sensing, it refers to the interaction between synaptic devices and external physical signals, which can control the expression of synaptic plasticity. Such an idea laid the foundation for artificial intelligence perception systems. The works shown in Table 1 reveal that external physic signals such as light, strain and temperature can effectively modulate the synaptic behaviors toward advanced neuromorphic sensing applications. These results demonstrate that the dynamics of synaptic plasticity can be realized by diversified modulation techniques, which promotes the implementation of more complex and diverse neuromorphic functions. It is obvious that research on plasticity modulation is currently in full swing, and a comprehensive summary of existing techniques is also needed to help researchers choose corresponding technical means according to specific application requirements. However, implementing multiple plasticity regulation techniques into a single device to achieve complex bio-inspired neuromorphic functions remains challenging.

**Table 1:** The summary of plasticity modulation techniques in synaptic devices

Targets	Functional element	Techniques	Achievements	Types	References
Controllable STP/LTP	Na^+^ intercalated WO_x_	Electrochemical intercalation	Improved LTP by 20 times	Chemical techniques	[28]
	2D Perovskite	Sn vacancies control	Transmission from LTP to STP	Chemical techniques	[29]
Optimized nonlinearity (NL)	Ta_2_O_5_	Oxygen vacancy control	Decreased NL from 4.61/− 8.1 to 1.21/− 0.15	Chemical techniques	[30]
	UVO-treated IGZO	Surface engineering	Decreased NL from 5.91/− 6.11 to 0.32/− 0.55	Chemical techniques	[31]
	WSe_2_/APTES/h-BN	Interface engineering	Decreased NL from 3.64/− 4.60 to 2.03/− 1.75	Chemical techniques	[32]
Improved symmetry	Nb-doped WSe_2_	Doing Strategies	Asymmetry ratio decreased from 0.97 to 0.54	Chemical techniques	[33]
Controllable STDP	WSe_2_ transistor	Dual-gate structure	Reconfigurable STDP	Device design	[34]
Controllable excitatory and inhibitory plasticity	Ferroelectric-gated CNT transistor	Dual-gate structure	Bi-directional response to stimuli	Device design	[35]
	h-BN/WSe_2_/BP	vdWs heterojunctions	Light-induced inhibitory plasticity	Device design	[36]
	Y_6_/PEA_2_SnI_4_	Hybrid channel	Light-induced inhibitory plasticity	Device design	[37]
Programmable synaptic behaviors	MoS_2_/h-BN/graphene floating-gate structure	vdWs heterojunctions	Switching between silent and function synapse	Device design	[38]
Visual perception	MoS_2_/QDs	Light-induced shift of V_th_	Retina-inspired neuromorphic sensing	External physic modulation	[39]
Tactile perception	Pressure sensor-Oscillator-Synaptic transistor	Convention from pressure to spike signals	Artificial afferent nerves and braille symbol recognition	External physic modulation	[40]
Temperature recognition	Chitosan-gated IGZO transistor	Temperature modulated plasticity	Temperature induced spiking AND to OR logic switching	External physic modulation	[41]

**Fig. 1:**
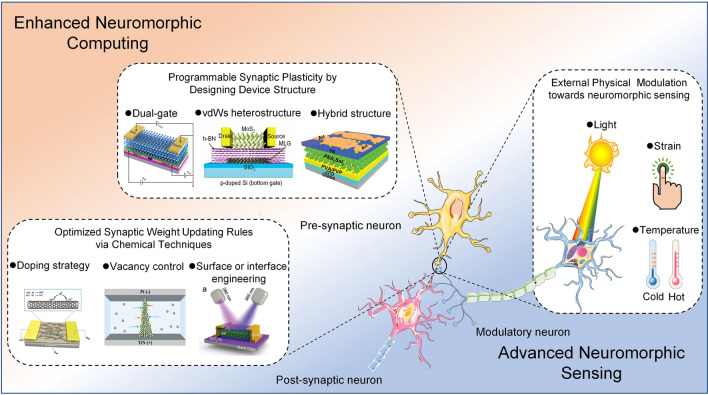
Overview of this review. Doping strategy [47]. Reproduced with Permission. Copyright 2021, Elsevier. Vacancy control [30]. Reproduced with Permission. Copyright 2022, Elsevier. Surface or interface engineering [95]. Reproduced with Permission. Copyright 2022, WILEY–VCH. Dual-gate [34]. Reproduced with Permission. Copyright 2020, The Author(s), under exclusive licence to Springer Nature Limited. vdWs heterostructures [38]. Reproduced under the terms of the Creative Commons CC BY license. Copyright 2022, The authors. Hybrid structure [37]. Reproduced with Permission. Copyright 2021, WILEY–VCH Verlag GmbH

Recently, neuromorphic devices have attracted much attention due to their ability to emulate synaptic plasticity, which aroused extensive discussion on the material synthesis and structural design for neuromorphic devices in the published review papers [5, 13, 43–46]. Although these works provide valuable insights into the design and fabrication of neuromorphic devices and the exploration of physical mechanisms and plasticity simulations, the increasing demand for functional diversity and integration of synaptic devices in the neuromorphic system is shifting the focus of research works from plasticity simulation to modulation, achieving better performance and complex functionality. Considering the lack of systematic analysis of plasticity modulation techniques, this review is dedicated to giving a comprehensive discussion on plasticity modulation techniques from the view of chemical techniques, device structure design and external physical modulation as shown in Fig. 1.

First, the chemical technique means such as surface modification and component regulation were discussed to modulate the properties of active materials in synaptic devices. The reason for choosing this topic is that chemical technique is an effective and simple way to directly modulate the properties of active materials, thereby changing the synaptic behaviors and optimizing the synaptic weight updating rules. Second, the designed device structures of artificial synapses are illustrated to clarify the principles of plasticity modulation. The emulation of synaptic plasticity indicates the non-volatile change of synaptic weight under stimulation, which depends on the memory mechanism of synaptic devices. In this way, the synaptic behaviors can be programmable by introducing different memory components through structure design such as floating-gate or ferroelectric gating. Third, the unique mechanisms of external physic modulation are clarified to show how the synaptic plasticity dynamically responds to external stimuli such as light, pressure or strain and temperature. The interaction between external physical signals and synaptic devices provides an opportunity to realize artificial perception systems. At last, the challenges and potential opportunities in neuroplasticity modulation techniques are proposed.

## Optimized Synaptic Weight Updating Rules via Chemical Techniques

Chemical techniques provide a featured way to tailor the properties of materials, promoting the design and fabrication of advanced functional devices. For example, He et al. achieved the polarity control of carbon nanotubes (CNTs)-based transistor by chemical doping strategy, where the p-type CNTs channel and n-type CNTs channel were fabricated by triethyl oxonium hexachloro antimonate doping and polyethylene imine doping, respectively [48]. In our previous work [29], we proposed the composition control on the ternary structure of two-dimensional perovskite, (PEA)_2_SnI_4_, to realize the dynamic transition from STP to LTP. In this section, several typical chemical techniques have been introduced to illustrate how to realize plasticity regulation from the perspective of materials science. The plasticity modulation by chemical techniques is mainly achieved through changes in the properties of active materials. Therefore, the active materials for neuromorphic devices should be sensitive to chemical doping or facilitating the property regulation during the material synthesis stage.

### Doping Strategy

**Fig. 2:**
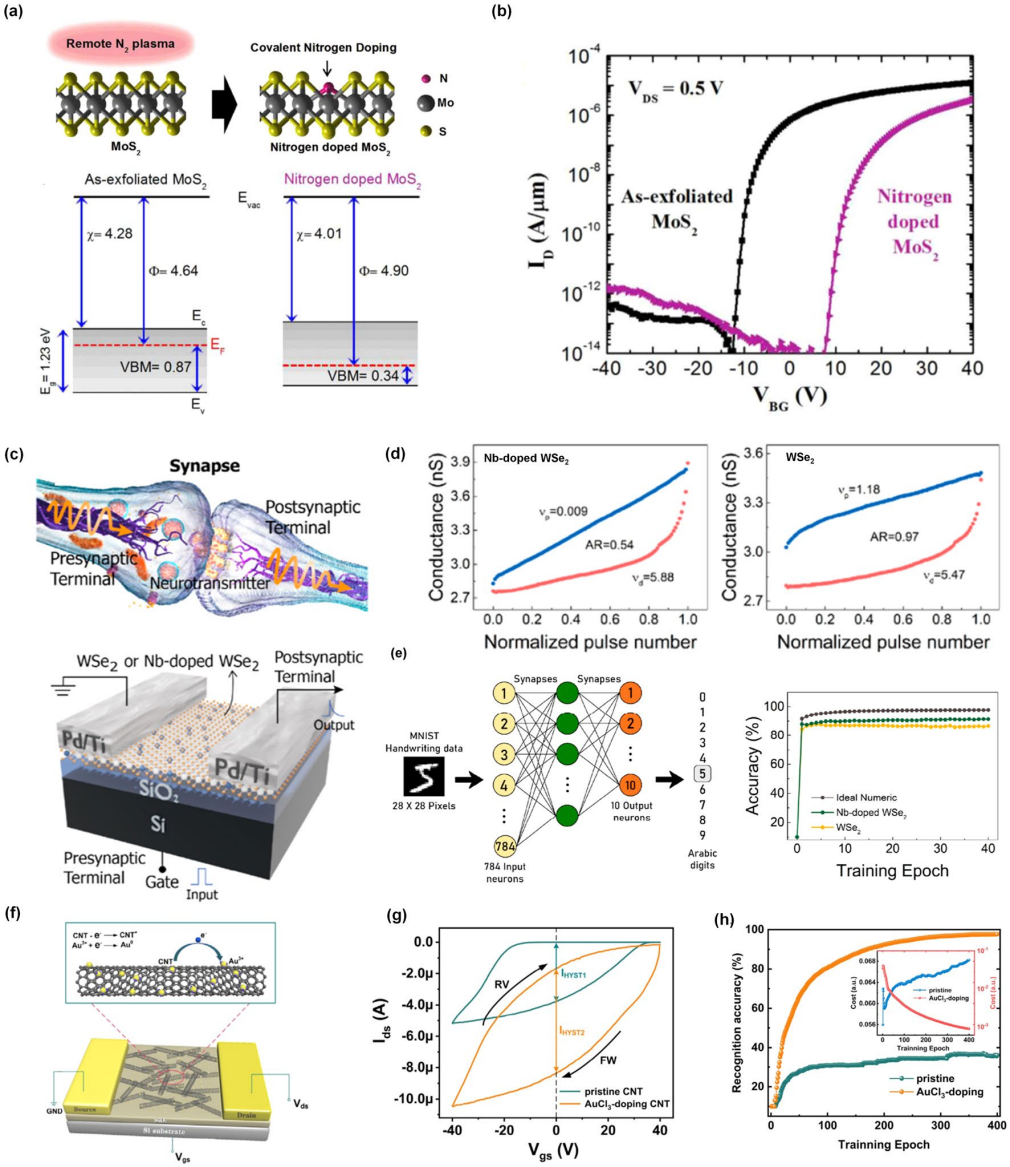
Chemical doping strategy for plasticity modulation. **a** Schematic of the covalent nitrogen doping in MoS_2_ upon N_2_ plasma surface treatment and energy band diagram for as-exfoliated MoS_2_ and nitrogen-doped MoS_2_. **b** Transfer curves (*I*_*D*_*-V*_*BG*_) of MoS_2_ transistor before and after nitrogen doping [61]. Reproduced with permission. Copyright 2016, American Chemical Society. **c** Three-dimensional diagram of a biological synapse and back-gate structured WSe_2_ or Nb-doped WSe_2_ transistor. **d** Conductance change under a train of pulses for the Nb-doped and pristine WSe_2_ synaptic transistor. **e** Schematic of an ANN based on backpropagation for pattern recognition accuracy based on the ideal networks, Nb-doped WSe_2_ and WSe_2_ synaptic devices [33]. Reproduced with permission. Copyright 2023, American Chemical Society. **f** Schematic illustration of the AuCl_3_-doped SWCNT FET. **g** Hysteretic loop of as-fabricated device before and after AuCl_3_ doping. Evolution of MNIST recognition accuracy as a function of training epoch for the pristine and AuCl_3_-doped CNFET synaptic device [47]. Reproduced with Permission. Copyright 2021, Elsevier

Chemical doping is an effective strategy to modify the electronic structures and properties of semiconductors, thus improving their performances in electronic or optoelectronic devices [49–52]. For synaptic devices, the characteristics of functional materials play an important role in their neuromorphic functions, especially considering the requirements of dynamic plasticity. Therefore, developing a specific doping strategy for plasticity modulation is of great importance to building artificial neuromorphic systems with tunable plasticity. Up to now, various doping strategies have been proposed to regulate and enrich the properties of semiconductors, including substitutional doping [53–55], surface charge transfer doping [56, 57] and intercalation [58–60]. Figure 2a illustrates an example of nitrogen-doped MoS_2_ by plasma treatment, where S atoms are partially replaced by N atoms, resulting in an increased work function, and providing evidence of p-type doping [61]. From Fig. 2b, the transfer curves of a back-gate transistor based on such an N-doped MoS_2_ channel present a positive shift of threshold voltage, further demonstrating the p-doping effect of nitrogen in MoS_2_. These results reveal that substitutional doping is an effective and stable way to modulate the electrical properties of semiconductors, especially for 2D materials, which also give a possible way to realize plasticity modulation.

Inspired by the high efficiency of the substitutional doping strategy, Azcatl et al. fabricated a synaptic transistor based on vanadium-doped monolayer MoS_2_ grown by in situ chemical vapor deposition, where the vanadium atom can trap/de-trap electrons, resulting in controllable hysteretic behaviors by changing the doping concentration [62]. In this work, the synaptic transistor based on highly vanadium-doped MoS_2_ exhibited the best synaptic potentiation and depression. Recently, an Nb-doped WSe_2_ back-gate transistor was developed to emulate the synaptic functions of biological ones as shown in Fig. 2c [33]. In this study, the incorporation of Nb introduced charge trap levels within the band gap and slowed down the degradation of trapped charges. As a result, it optimized artificial synaptic plasticity by promoting enhanced short-term and long-term plasticity, increased multilevel states as well as improved symmetry ratios. From Fig. 2d, the values of NL have been greatly decreased from 1.18 (undoped) to 0.009 (Nb-doped) and the values of asymmetry ratio were evaluated to be 0.97 (undoped) and 0.54 (Nb-doped), respectively, which contributed to a better recognition accuracy of artificial neural networks (ANNs) (Fig. 2e). Thus, it can be inferred that chemical doping provides a stable and controllable way to modulate the synaptic plasticity for the development of excellent neuromorphic computing architectures.

Another widely used doping strategy is surface charge transfer doping, which is an effective and non-destructive doping technique, especially for 2D semiconductors [63]. The carrier charge transfer between the surface dopant and semiconductor can induce reliable doping in a non-destructive manner, which can modulate the carrier concentration in semiconductors. In our previous work [47], we proposed an opposite strategy to enhance the p-type conducting behavior of the SWCNT channel by AuCl_3_ doping due to the capture of the electrons in the SWCNT channel by Au^3+^ (Fig. 2f). Benefiting the improved conductance of the SWCNT channel (Fig. 2g), both excitatory and inhibitory synaptic behaviors were enhanced with an improved symmetry, which contributed to an increased recognition accuracy (~ 98%) for the recognition of handwritten digits (Fig. 2h). It is noted that the high NL or low symmetry ratios has been attributed to unipolar conducting behaviors of semiconductor channel because potentiation and depression have been usually achieved by the pre-synaptic voltages with the opposite polarity. It means that for p-type transistors, a negative voltage made the channel be switched “ON” but a positive one contributed to the pinching of the channel, resulting in the difference in available conductive states and their updating speed, thus the poor symmetry ratios between potentiation behavior and depression behavior. To solve this issue, surface charge transfer doping is an effective way to reduce the NL and improve the symmetry of synaptic devices because it could easily tune the conductive states, even conductive polarity, of semiconductor channels [64–66].

Besides the above common doping strategies, the electrochemical intercalation of foreign species at the interface between atomically thin van der Waals (vdWs) layered structures has attracted much attention due to its excellent tunability of electronic structures [67]. Generally, electrochemical intercalation has been utilized to emulate the synaptic plasticity in an ion-gated transistor by exactly controlling the concentration of Li or Na ions in host materials [68, 69]. However, the synaptic functions were affected by the high ionic diffusivity, resulting in poor retention stability. Recently, Lee et al. proposed an all-solid-state Na ion-based synaptic transistor with a WO_x_ channel with better state retention than Li ion-based ones due to the lower ionic diffusivity of Na ions in WO_x_ [28]. Baek et al. reported a two-terminal Au/Li_x_CoO_2_/Pt artificial synaptic device, which exhibited extraordinary neuromorphic behaviors based on a progressive dearth of Li in Li_x_CoO_2_ film [70]. The improved weight control functionality was realized by precisely controlling the intercalation and deintercalation of Li-ion inside the films. It can be inferred that the synaptic behaviors can be also effectively modulated by the concentration and type of ions and their special interactions with semiconductors.

### Vacancy Control

Vacancies are formed when atoms escape from the lattice, which has been widely observed in metallic oxides [71, 72], nanostructured semiconductors [73] and atom-thick 2D solids [74]. The existence of vacancies could induce changes in the materials’ electronic and geometric structures, offering a potential opportunity to manipulate the physicochemical properties of materials by tuning the concentration, types, and distribution of vacancies [75, 76]. As one of the typical neuromorphic devices, two-terminal memristors that directly relate electrical charge to flux has been developed to achieve synaptic functions such as PPF, STP and LTP [77]. The resistive switching mechanism of memristors based on metallic oxides has been widely attributed to the migration, accumulation, and rearrangement of oxygen vacancies within a dielectric medium driven by the external electric field [78]. Based on oxygen vacancy engineering, the adjustable electric conductivity of resistive switching layers with memory characteristics can be used to represent the connection strength between neurons, namely synaptic weight [79–81]. Specifically, manipulating the oxygen vacancies in the resistive switching layers could directly modulate the synaptic behaviors, resulting in dynamic plasticity.

**Fig. 3:**
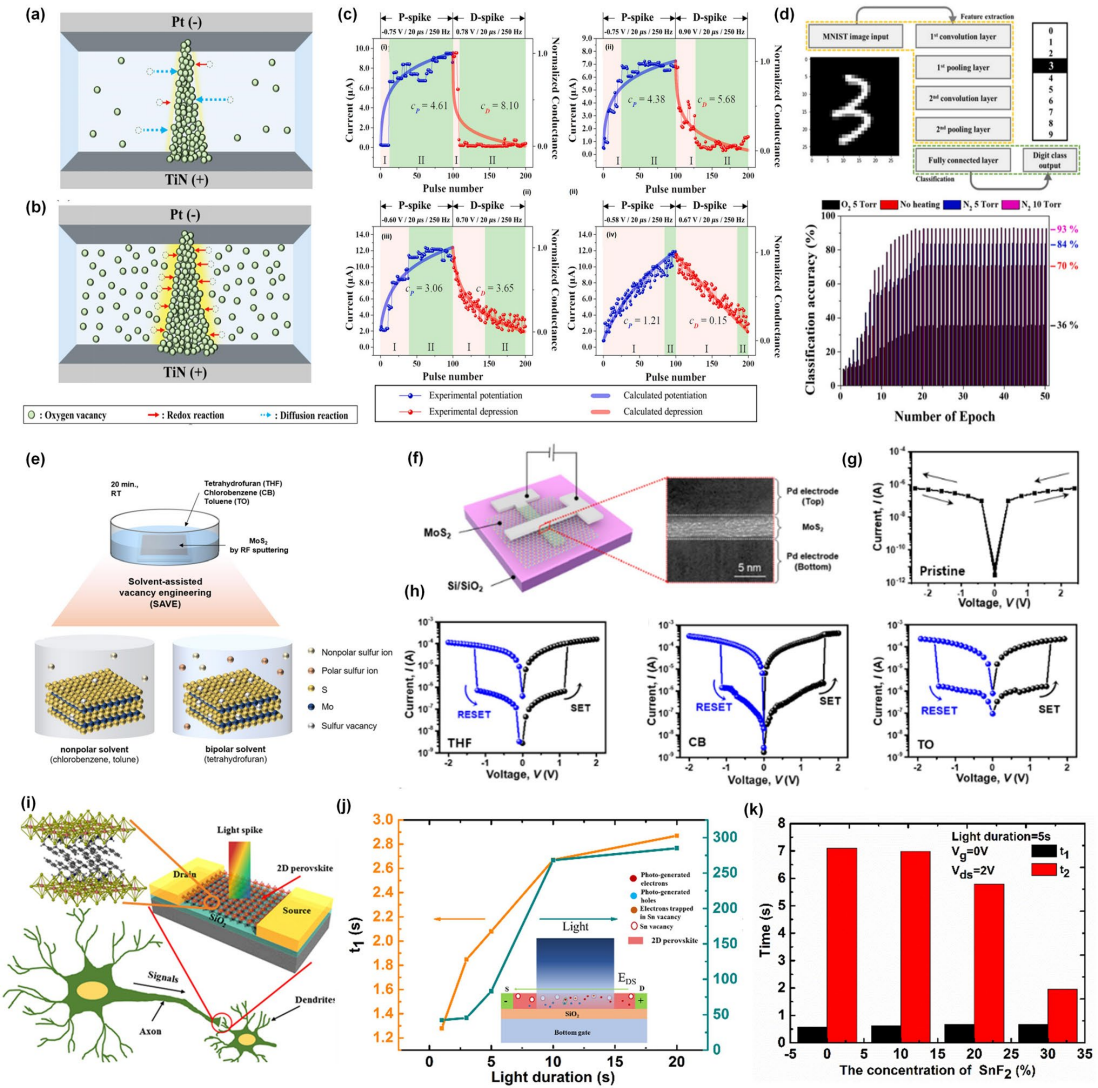
Chemical doping strategy based on the element control. **a** and **b** Schematic of oxygen vacancies and growth of the filament. **c** Variation of the conductance triggered by 100 potential spikes and 100 depression spikes for Ta_2_O_5_ memristors heated under different atmospheres: (i) O_2_ at 5 Torr, (ii) without heating, (iii) N_2_ at 5 Torr, and (iv) N_2_ at 10 Torr. **d** Schematic of the CNN structure and calculated recognition accuracy of the MNIST patterns with respect to the number of training epochs under various atmospheres [30]. Reproduced with Permission. Copyright 2022, Elsevier. **e** Solvent-assisted vacancy engineering (SAVE) method with MoS_2_. **f** A schematic of the MoS_2_ device structure and cross-sectional transmission electron microscopy (TEM) image. **g** *I–V curve* of pristine-MoS_2_ without the memristive effect. **h** *I–V* curves of tetrahydrofuran (THF)-MoS_2_, chlorobenzene (CB)-MoS_2_, and toluene (TO)-MoS_2_ with bipolar resistive switching [85]. Reproduced with Permission. Copyright 2023, Royal Society of Chemistry. **i** Schematic of a biological synapse and synaptic device based on 2D perovskite. **j** The calculated two distinct time constants (t_1_ and t_2_) with a function of duration time of light spikes. **k** The time constants t_1_ and t_2_ are plotted as a function of the concentration of the added SnF_2_ [29]. Reproduced with Permission. Copyright 2019, WILEY–VCH

As shown in Fig. 3a, b, Hwang et al. fabricated a Ta_2_O_5_ memristor with bipolar switching properties based on the growth and destruction of oxygen vacancy filaments [30]. In this work, the authors proposed a simple annealing strategy under different various atmospheres to improve the conductance modulation linearity of synaptic devices. The X-ray photoelectron spectroscopy analysis indicated that numerous oxygen vacancies were formed in the Ta_2_O_5_ layer when heated under N_2_ at 10 Torr. The increase of oxygen vacancy could contribute to the fast redox reaction, which further controlled the growth of oxygen vacancy filaments and improved the conductance modulation linearity. From Fig. 3c, the best conductance modulation linearity was achieved with the smallest curvatures of the potentiation (*C*_*p*_) and depression (*C*_*d*_) curves of the Ta_2_O_5_ memristors heated under N_2_ at 10 Torr (iv) compared with other conditions. Benefiting from the good linearity, the Ta_2_O_5_ memristors heated under N_2_ at 10 Torr exhibited the highest classification accuracy of the convolutional neural network for the learning process as shown in Fig. 3d. This work illustrated how to achieve plasticity modulation by applying different annealing conditions to control the growth and migration of oxygen vacancies without the insertion of extra layers. This simple method is very suitable for the oxide-based memristors for neuromorphic chips.

Moreover, 2D materials-enabled memristors also exhibit great potential in neuromorphic applications because their properties can be precisely controlled by introducing the defects, such as vacancies, in atomic structures during the material synthesis stage or by surface plasma treatment [82–84]. Recently, a novel solvent-assisted vacancy engineering (SAVE) method was proposed to modulate sulfur vacancies in MoS_2_, avoiding the physical damage on the structure of MoS_2_ by the ion bombardment (Fig. 3e) [85]. This method contributed to the non-destructive and uniform generation of sulfur vacancies on the MoS_2_ surface, which could be further implemented in a synaptic memristor with a sandwich structure (Fig. 3f). Compared with pristine MoS_2_-based memristors (Fig. 3g), the memristors based on SAVE-treated MoS_2_ exhibited non-volatile characteristics, which also depended on the types of solvent. In this work, the THF-MoS_2_ synaptic memristor showed high uniformity and reliability and a much higher recognition accuracy than that of pristine MoS_2_ by Modified National Institute of Standards and Technology (MNIST) training. The underlying mechanism for such a plasticity modulation technique based on solvent engineering could be attributed to the fact that when solvents have similar Hansen solubility parameters, the bipolar solvent could generate sulfur vacancies because it can remove both polar and nonpolar sulfur, resulting in sulfur vacancies.

Another way to regulate the concentration of vacancies in nanomaterials is to add control agents during the synthesis phase. This idea has been well implemented in solution-processable 2D layered perovskites, which provided an opportunity to manipulate its physical properties by introducing specific ions. For example, a vertically aligned 2D halide perovskite-enabled artificial synapse was demonstrated, where the nanostructure of perovskite grains was adjusted by employing the pseudo-halide SCN additive, thus achieving the programmable potentiation and depression with distinguished multistates [86]. In another work, 2D layered perovskite ((PEA)_2_SnI_4_) was chosen as a conductive channel for a two-terminal synaptic device to emulate the light-stimulated synaptic behaviors (Fig. 3i) [29]. In this work, the photo-induced memory characteristics were attributed to two different trapping states: the shallower traps induced by the uncompensated dangling bonds or the structural defects and the deeper traps induced by Sn vacancies. The photocurrent decay curves were fitted by a temporal model with two exponential terms, the smaller *t*_*1*_ for shallower traps and the larger *t*_*2*_ for deeper traps, respectively (Fig. 3j). Based on this principle, we further proposed a component regulation technology by introducing SnF_2_ to control the amount of Sn vacancies, resulting in the adjustable value of *t*_*2*_ (Fig. 3k). In general, the larger *t*_*2*_ means a stronger memory effect and is responsible for the LTP. Therefore, by controlling the concentration of SnF_2_, the conversion between STP and LTP could be manipulated to achieve dynamic plasticity.

### Surface or Interface Engineering

Electronic devices are usually constructed by various types of materials, resulting in the specific surface or interface between them, especially for 2D materials, where the intrinsic atomic level thickness makes its properties extremely susceptible to surface or interface qualities [87]. The impact of surface or interface characteristics on the performance of two-dimensional electronic devices can be divided into the following aspects:

i) Contact resistance: In electronic devices, interfaces play a crucial role in the electrical contact between different materials. The presence of high contact resistance at the interface can hinder efficient charge carrier transport, leading to increased power consumption, reduced device performance, and limited functionality [88]. Optimizing the interface properties can help reduce contact resistance and improve device performance.

ii) Charge carrier scattering: Interfaces or surfaces can introduce scattering centers that scatter charge carriers (electrons or holes) as they move through the device. This scattering can degrade the mobility of charge carriers, reducing the overall device performance. By engineering interfaces with appropriate properties, such as reduced defects or proper passivation, the scattering can be minimized, leading to enhanced charge carrier mobility and improved device performance [89].

iii) Band alignment and energy level alignment: Interfaces between different materials can lead to the formation of energy barriers or energy level mismatches [90]. These energy barriers can impede charge carrier injection or extraction at the interfaces, affecting the device's efficiency and overall performance. By carefully designing the interface properties, such as adjusting the energy levels or achieving proper band alignment, efficient charge carrier injection and extraction can be achieved, improving device performance.

iv) Surface passivation and protection: 2D materials, such as graphene or TMDs, have exposed surfaces that can be sensitive to environmental factors, such as moisture or oxygen [91]. These interactions can degrade the material's properties and affect device performance. By introducing suitable surface passivation or protection layers at the interfaces, the materials can be shielded from external factors, preserving their properties, and maintaining device performance over time.

**Fig. 4:**
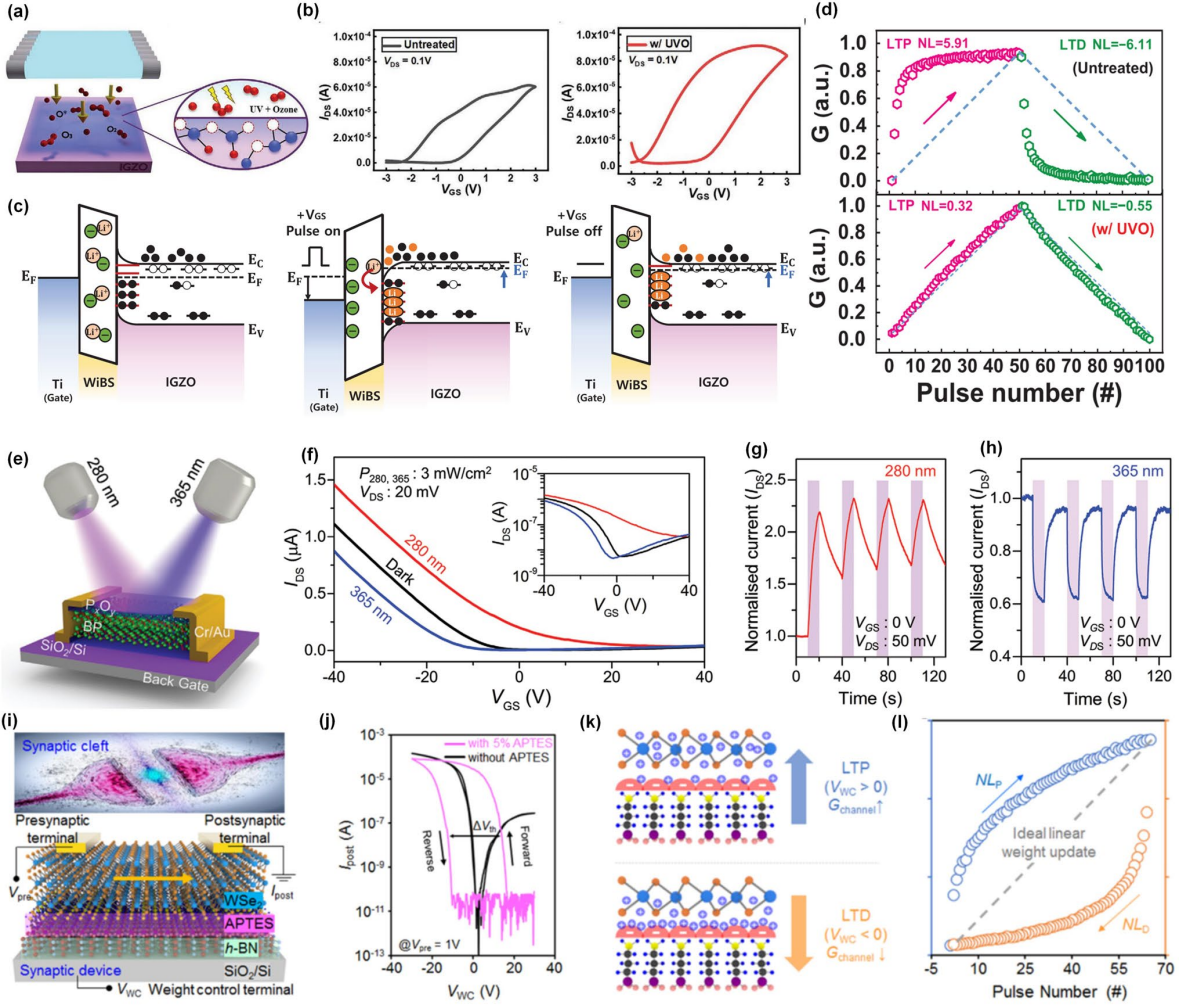
Chemical modulation strategies based on surface or interface engineering. **a** Schematic illustration of chemical bonding at the surface of UVO-treated IGZO film. **b** Hysteretic loops of the IGZO-based transistors without UVO treatment and after UVO treatment. **c** Energy band diagram of as-fabricated synaptic transistors and the illustration of carrier trapping/de-trapping through the interface. **d** Comparison of linearity and symmetricity between untreated and UVO-treated (w/UVO) devices [31]. Reproduced with Permission. Copyright 2022, WILEY–VCH. **e** Schematic of a black phosphorus-based transistor with the oxidized surface. **f** Transfer curves of such devices in dark and under illumination of 280 and 365 nm. The normalized transient photocurrent under the illumination of **g** 280 nm and **h** 365 nm, respectively [95]. Reproduced with Permission. Copyright 2022, WILEY–VCH. **i** The schematic illustration of an WSe_2_-based synaptic device with APTES modified interface. **j** Hysteretic behaviors of WSe_2_-based synaptic devices with and without APTES layer. **k** Schematic illustrations on hole releasing and trapping mechanisms. **l** Nonlinearity extracted in the LTP/LTD characteristic curves [32]. Reproduced with Permission. Copyright 2020, American Chemical Society

It can be inferred that the properties of interfaces or surfaces in electronic devices, especially in devices based on 2D materials, have a significant impact on device performance. Optimizing these surfaces or interfaces can help modulate the electrical or optical properties of 2D materials to realize plasticity modulation. As shown in Fig. 4a, an ultraviolet ozone (UVO) treatment was applied to functionalize the surface of indium-gallium-zinc oxide (IGZO) with trap sites, which resulted in the enlarged hysteresis loop in the transfer curves due to the interaction between trap sites and lithium cations in electrolytes (Fig. 4b) [31]. Under positive pulse, the lithium cations could occupy the trap sites induced by UVO treatment, liberating originally trapped electrons into IGZO channel to maintain the high current value even after the pulse removed (Fig. 4c). Benefitting from the adjustability on the channel conductance by the number and polarity of the input voltage spikes, the UVO-treated synaptic devices exhibited a lower NL of long-term potentiation (LTP) and depression (LTD) compared with untreated one (Fig. 4d). This work demonstrated that the surface engineering could be an efficient technique for sophisticated ion transport and enabled various applicability of electrolyte-gated synaptic transistors with tunable plasticity.

As mentioned above, surface passivation is another popular technique to protect 2D materials from damage of the surrounding environment. In fact, surface passivation can not only protect materials from structural or performance damage, but also modify the electrical or optical properties of materials. Specifically, few-layer black phosphorus (BP) flakes have been found to degrade rapidly in ambient conditions due to a photo-assisted oxidation reaction with oxygen dissolved in the water [92]. However, recent studies revealed that the oxidation of BP into phosphorus oxide (P_x_O_y_) can serve as a self-passivation layer for the underneath BP and improve the performance of BP-based electronic devices [93, 94]. As shown in Fig. 4e, Ahmed et al. demonstrated a fully light-controlled neuromorphic transistor based on layered BP flakes with an amorphous thin layer of native P_x_O_y_ on their surfaces [95]. Interestingly, such devices showed a wavelength-dependent photoresponse with a positive shift of threshold voltage after the illumination of 280 nm and a negative shift after the illumination of 365 nm (Fig. 4f). From Fig. 4g, h, it is obvious that a negative photocurrent could be achieved under the illumination of 365 nm, which has been attributed to the carrier scattering by charged defects under low energy (365 nm) excitation. In this way, all-optically driven neuromorphic computation is demonstrated by machine learning to classify numbers and recognize images based on high energy (280 nm) induced potentiation and low energy (365 nm) induced depression behaviors.

Besides, 2D layered materials with no dangling bonds and diverse band structures can be stacked to construct the devices with different functions, where the interface between layered structures can be utilized to regulate the electrical performances. Seo et al. have realized dynamic synaptic plasticity in an optic-neural synaptic device by inserting a weight control (WC) layer on the defect-free interface between h-BN and WSe_2_ to precisely control the channel conductance [96]. As shown in Fig. 4i, a WC layer composed of 3-aminopropyltriethoxysilane (APTES) has been inserted on the interface between WSe_2_ and h-BN, which dominated the hysteresis behaviors in the transfer curves (Fig. 4j) [32]. A bottom-gate terminal could work as a weight control terminal by applying a voltage pulse to manipulate the holes trapping and de-trapping at the APTES interface, resulting in the conversion between LTP and LTD (Fig. 4k) and an almost ideal NL (Fig. 4l).

Overall, after reviewing the prevailing chemical techniques for plasticity modulation, it can be inferred that chemical techniques were an effective way to achieve dynamic plasticity by directly modulating the properties of 2D materials or their heterostructures. The underlying mechanism was mostly attributed to the controllability of the carrier or charge trapping/de-trapping process, which further affected the synaptic weight updating rules and non-volatile memory characteristics. However, it is also noted that chemical techniques cause permanent changes in the properties of materials or the physical structure of devices. For example, for the doping strategy, once the type and concentration of dopants were determined, the function of synaptic devices was fixed. For synaptic devices that expect programmable operation, chemical doping technology may lack flexibility and reconfigurable characteristics.

## Programmable Synaptic Plasticity by Designing Device Structure

**Fig. 5:**
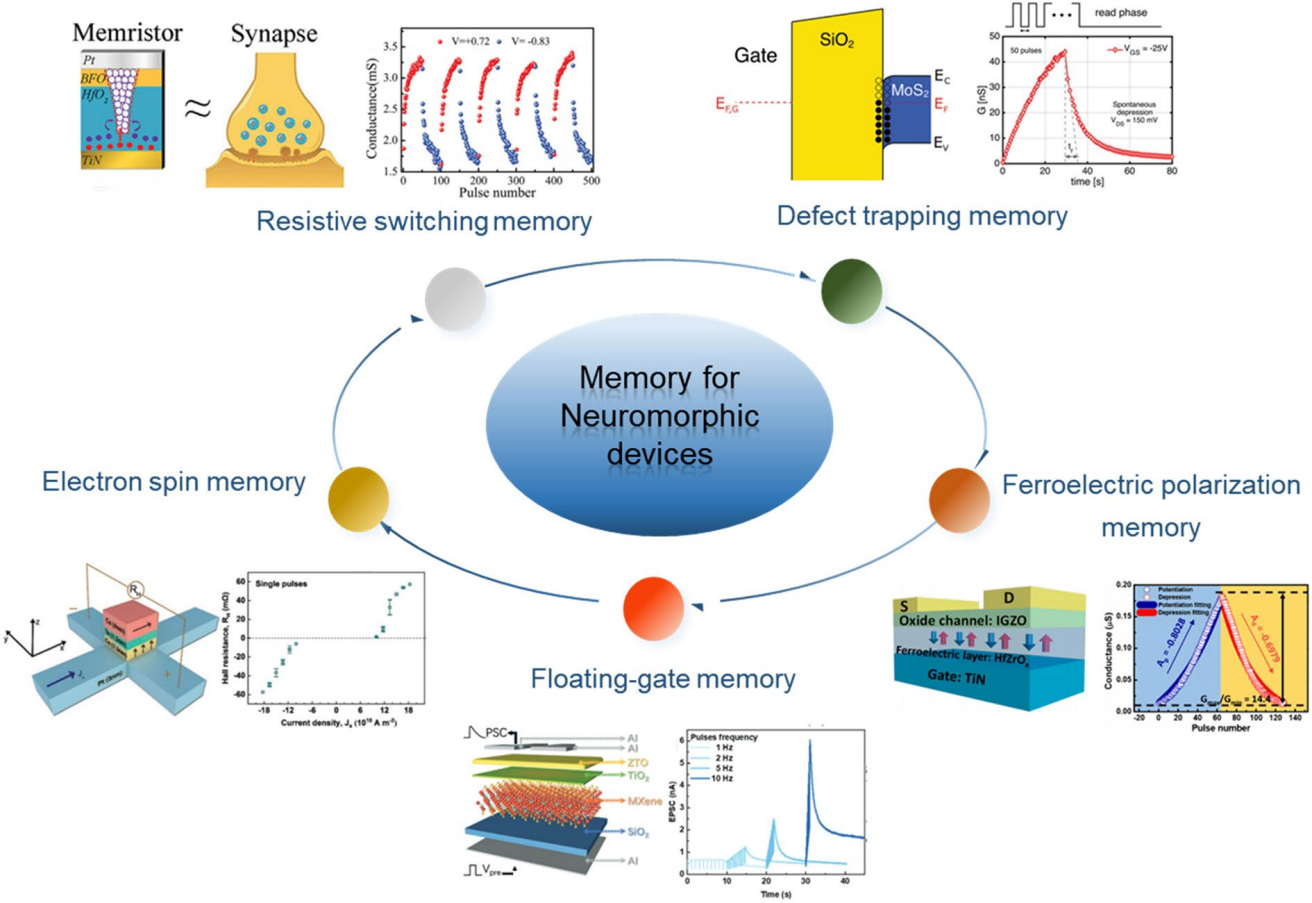
Different memory mechanisms for neuromorphic devices. Defect trapping memory [99]. Reproduced with Permission. Copyright 2022, The authors. Ferroelectric polarization memory [24]. Reproduced with Permission. Copyright 2019, American Chemical Society. Floating-gate memory [100]. Reproduced with Permission. Copyright 2021, WILEY–VCH. Electron spin memory [101]. Reproduced with Permission. Copyright 2021, WILEY–VCH. Resistive switching memory [102]. Reproduced with Permission. Copyright 2020, WILEY–VCH

To overcome the limitation of chemical techniques, designing the device structures to control the expression of synaptic plasticity could be a more advantageous choice. In recent years, great efforts have been devoted to designing and optimizing device structures for the diversification of device performance, such as nBn or pBp structures for unipolar barrier photodetectors [97], double-gate floating structures for enhanced non-volatile memory [21], and mixed-dimensional vdWs heterostructures for reconfigurable optical memory [98]. In biological systems, synapses are the basic functional units that simultaneously achieve both information processing and storage through plasticity modulation. Therefore, the manipulation of memory behaviors provides a feasible way to achieve plasticity modulation by device structure design. Figure 5 shows the prevailing memory mechanisms and their device structures for neuromorphic devices [24, 99–102]. Integrating different memory mechanisms into a single device or manipulating different storage states in a single memory device to control the response to pre-synaptic stimuli makes it possible to realize programmable or reconfigurable synaptic plasticity. In this section, we would like to discuss several ingenious device designs for realizing plasticity modulation and comment on their working principles.

### Dual-Gate Structure

**Fig. 6:**
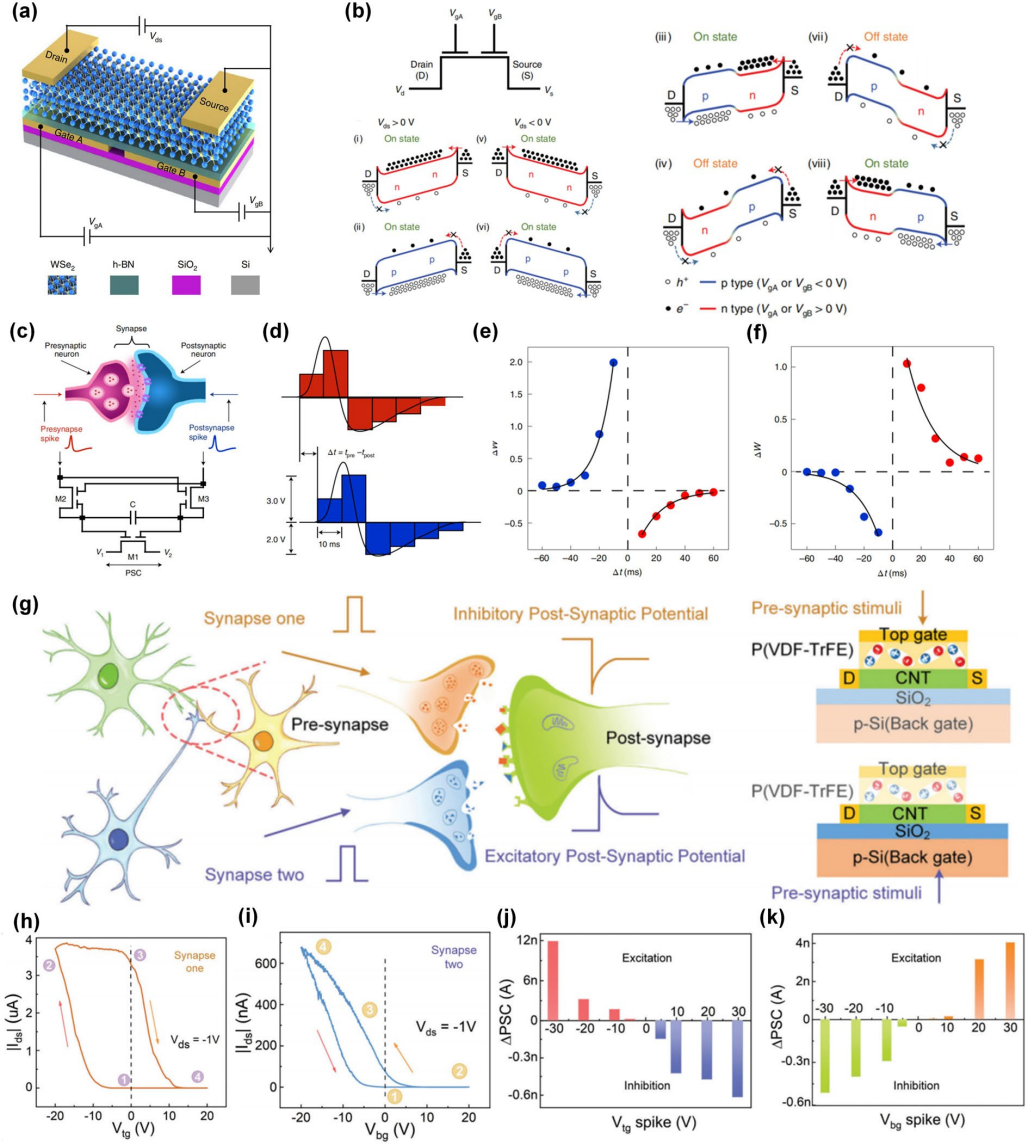
Dual-gate synaptic transistor with modulated plasticity. **a** Schematic of WSe_2_-based synaptic transistors with dual bottom-gate. **b** Energy level diagram of the WSe_2_ channel under different combination of gate voltages. **c** Schematic of a biological synapse (top) and a circuit for reconfigurable synaptic functions (bottom). **d** Illustration of six rectangular waves to simulate presynaptic (red) and post-synaptic (blue) spikes. The STDP learning rules for **e** anti-Hebbian (V_1_ = 0 V, V_2_ = 3 V) and **f** Hebbian (V_1_ = 3 V, V_2_ = 0 V) [34]**.** Reproduced with Permission. Copyright 2020, The author(s), under exclusive licence to Springer Nature Limited. **g** Schematic diagram of synaptic transmission between neurons and the operation mode in a dual-gate artificial synaptic transistor. **h** Clockwise hysteretic behavior measured from ferroelectric top-gate transistor. **i** Anti-clockwise hysteretic behavior measured from SiO_2_ bottom-gate transistor. **j** Change of PSC (ΔPSC) induced by different top-gate voltages. **k** Change of PSC (ΔPSC) induced by different bottom-gate voltages [35]. Reproduced with Permission. Copyright 2023, WILEY–VCH

In biological synapses, modulatory neurons could refresh the connection strengths between two other neurons and control the neural signal transmissions through the synapses, which contributes to the implementation of complex neural behaviors [103, 104]. Therefore, multiterminal transistors give the ability to modulate the electrical performance by controlling the polarity of voltages applied to the gate or drain electrodes. It means that the functions of such devices can be easily manipulated by different voltage configuration combinations, which is also well-known as reconfigurable operation. As shown in Fig. 6a, Pan et al. fabricated a homojunction device made from 2D WSe_2_ with a dual bottom gate, Gate A and Gate B, to control the electrical characteristics by polarity combinations of the gate and drain voltage inputs [34]. From Fig. 6b, the polarity of *V*_*gA*_ and *V*_*gB*_ determined the injection of carriers by modulating the height of the Schottky barrier between WSe_2_ and drain/source electrodes, while the polarity of *V*_*ds*_ determined the on/off state of the conductive channel. Therefore, eight different working operating principles were achieved, which was the foundation of reconfigurable multifunctional logic and neuromorphic capabilities. The authors further proposed synaptic circuits based on only three such homojunction devices to achieve reconfigurable spiking-timing-dependent plasticity (STDP) (Fig. 6c). Here, they used a combination of six rectangular wave pulses to simulate the pre- and post-synaptic spikes (Fig. 6d). Figure 6e shows the STDP behaviors at V_1_ = 0 V and V_2_ = 3 V, which resembled the anti-Hebbian synaptic learning rule with enhanced synaptic weight when Δt < 0. While by varying the relative electrical potential between V_1_ and V_2_ (V_1_ = 3 V and V_2_ = 0 V), the STDP behaviors indicated a Hebbian synaptic learning rule that indicated an opposite response (Fig. 6f). In this case, synaptic weight updating rules could be switched by changing the polarity combinations of the dual-gate voltages, which referred to the reconfigurable synaptic functions.

Another advantage of dual-gate structure is introducing different dielectric layers at different gate terminals to integrate diverse synaptic functions into a single device. In a recently published work [35], a multiterminal synaptic transistor with ferroelectric top-gating and SiO_2_ bottom-gating has been designed and fabricated to emulate the bi-directional modulation on synaptic plasticity (Fig. 6g). Due to the opposite direction of hysteretic loops in transfer curves by ferroelectric top-gating (Fig. 6h) and SiO_2_ bottom-gating (Fig. 6i), such device showed opposite response to the same pre-synaptic voltages. From Fig. 6j, positive synaptic weight change could be achieved for negative *V*_*tg*_ spikes, indicating excitatory synaptic plasticity and negative synaptic weight change referring to inhibitory synaptic plasticity was obtained for positive *V*_*tg*_ spikes. By switching the input terminal, inhibitory synaptic plasticity was achieved for negative *V*_*bg*_ spikes while excitatory synaptic plasticity was obtained for positive *V*_*bg*_ spikes. Such bi-directional modulation on plasticity was attributed to two different physical mechanisms, namely ferroelectric polarization switching in top-gate and carrier trapping/de-trapping process through the interface defects. Based on such operation principles, the conversion of inhibitory and excitatory synaptic plasticity can not only be achieved by changing the polarity of pre-synaptic voltages but switching the input terminal, which opened a new way to fulfill diversified plasticity modulation techniques in a single device.

### vdWs Heterostructure

Since graphene with a single carbon atom-thick planar structure was obtained by mechanical exfoliation [105], 2D materials have gradually entered the vision of researchers and promoted the development of 2D electronics. In the last few decades, the 2D material family has achieved tremendous prosperity, covering semiconductors [106], insulators [107], and metals [108], which are the essential components that constitute electronic devices. Besides, it is fascinating that such dangling-bond-free atomic planes can also be stacked layer by layer into designer heterostructures in a precisely chosen sequence to form vdWs heterostructures like “Lego Blocks”, bringing unusual properties and new phenomena [109]. Therefore, utilizing van der Waals heterojunction design to achieve plasticity modulation is a promising approach.

**Fig. 7:**
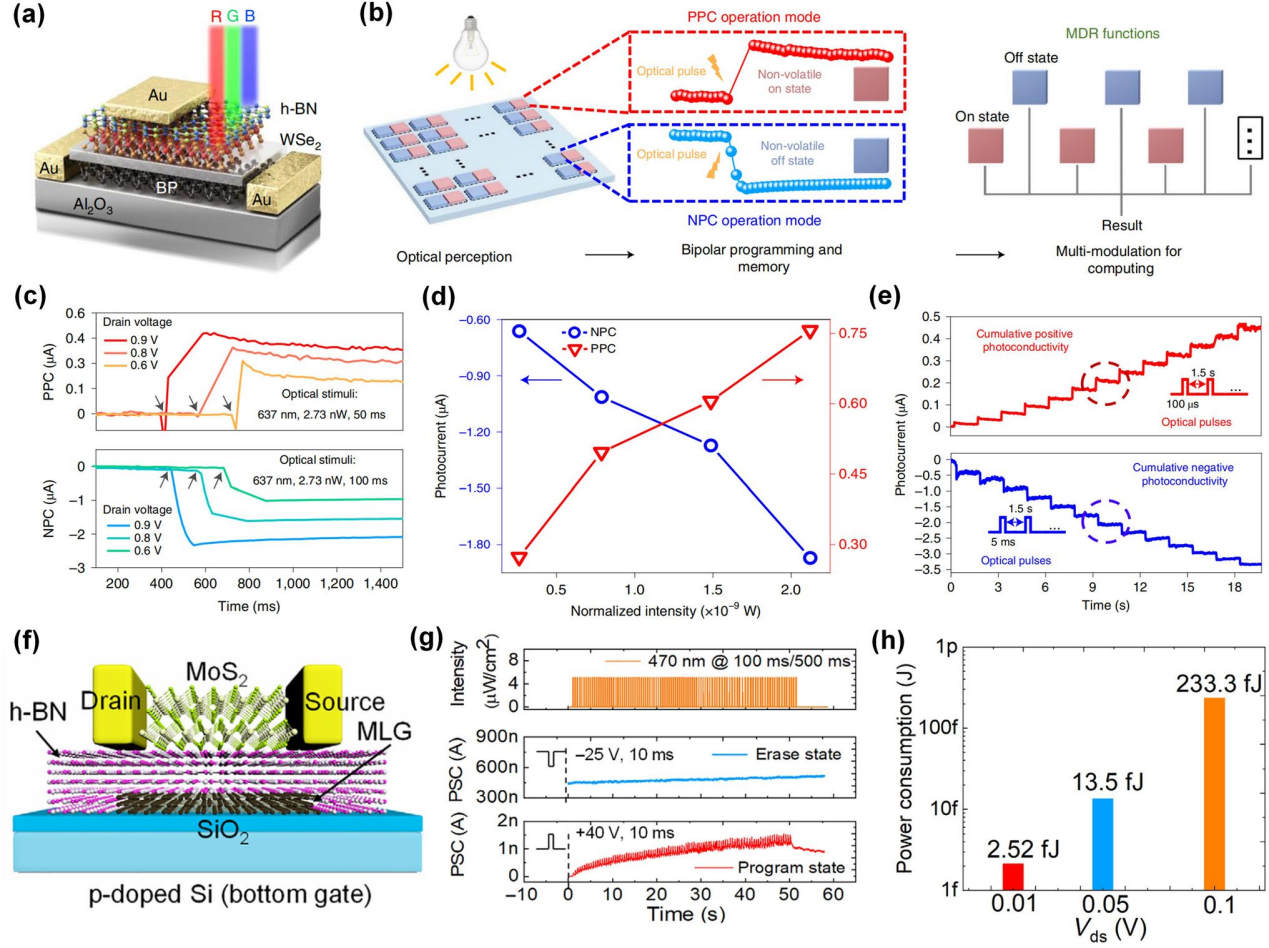
vdW heterostructures enabled synaptic transistors. **a** Schematic of h-BN/WSe_2_/BP vdWs heterojunction-based transistor. **b** Retina-inspired all-in-one 2D retinomorphic devices and the optical perception and response of the 2D materials mimic the signal collection and conversion of the photoreceptors. **c** Essential PPC and NPC curves under drain voltage modulation. **d** Modulation of photocurrent by normalized laser intensity. **e** Cumulative positive and negative photoconductivity with progressive multilevel states [36]. Reproduced with Permission. Copyright 2022, The Author(s), under exclusive licence to Springer Nature Limited. **f** Devices structure of graphene/h-BN/MoS_2_ vdWs heterojunction-based floating-gate transistor. **g** Electrical response to the light stimuli at program state and erase state. **h** Power consumption as a function of *V*_*ds*_ [38]. Reproduced under the terms of the Creative Commons CC BY license. Copyright 2022, The authors

In this field, Peng Zhou’s group has developed a series of van der Waals heterojunction devices and verified their potential applications in various fields such as photodetectors [97], floating-gate memory [110], and reconfigurable logic-in-memory devices [111]. In 2022, they proposed an all-in-one retinomorphic hardware device based on 2D vdWs heterostructures as shown in Fig. 7a, which integrated the perception, memory and computing capabilities for the detection and recognition of moving trolleys into a single device [36]. The implementation of this concept mainly relied on progressively tunable positive/negative photoresponses with non-volatile memory characteristics (Fig. 7b–e). The underlying mechanism for positive photocurrent (PPC) and negative photocurrent (NPC) was attributed to the gate-voltage programmable carrier tunneling process through vdWs interfaces. A similar working operation has been also implemented in a vdWs heterostructure-enabled floating-gate synaptic transistor with multilayer graphene as the floating-gate, h-BN as tunneling layer, and MoS_2_ as a conductive channel (Fig. 7f) [38]. This work demonstrated a programmable operation to control the response to pre-synaptic light stimuli. As shown in Fig. 6g, when a negative bottom-gate pulse was applied, such a float-gating transistor was set to an “erase state” with high conductance due to the electrons tunneling from graphene to the MoS_2_ channel, which suppressed the response to light stimuli, resulting in a silent synapse. After a positive voltage pulse was applied, the MoS_2_ channel would be switched to a “program state” with ultra-low conductance due to the electrons tunneling from the MoS_2_ channel to graphene and exhibit the response to light stimuli in an excitatory synapse-like way. The working mode could be switched from “Silent” to “Exhibitory” by applying different polarity of bottom-gate voltages. Benefitting from the suppressed background current at the program state, an ultra-low power consumption of ~ 2.52 fJ per light spike event was achieved. These results demonstrated that manipulating the carrier transport characteristics through the designed vdWs heterostructures could be an efficient way to realize plasticity modulation in a programmable way.

### Hybrid Structure

**Fig. 8:**
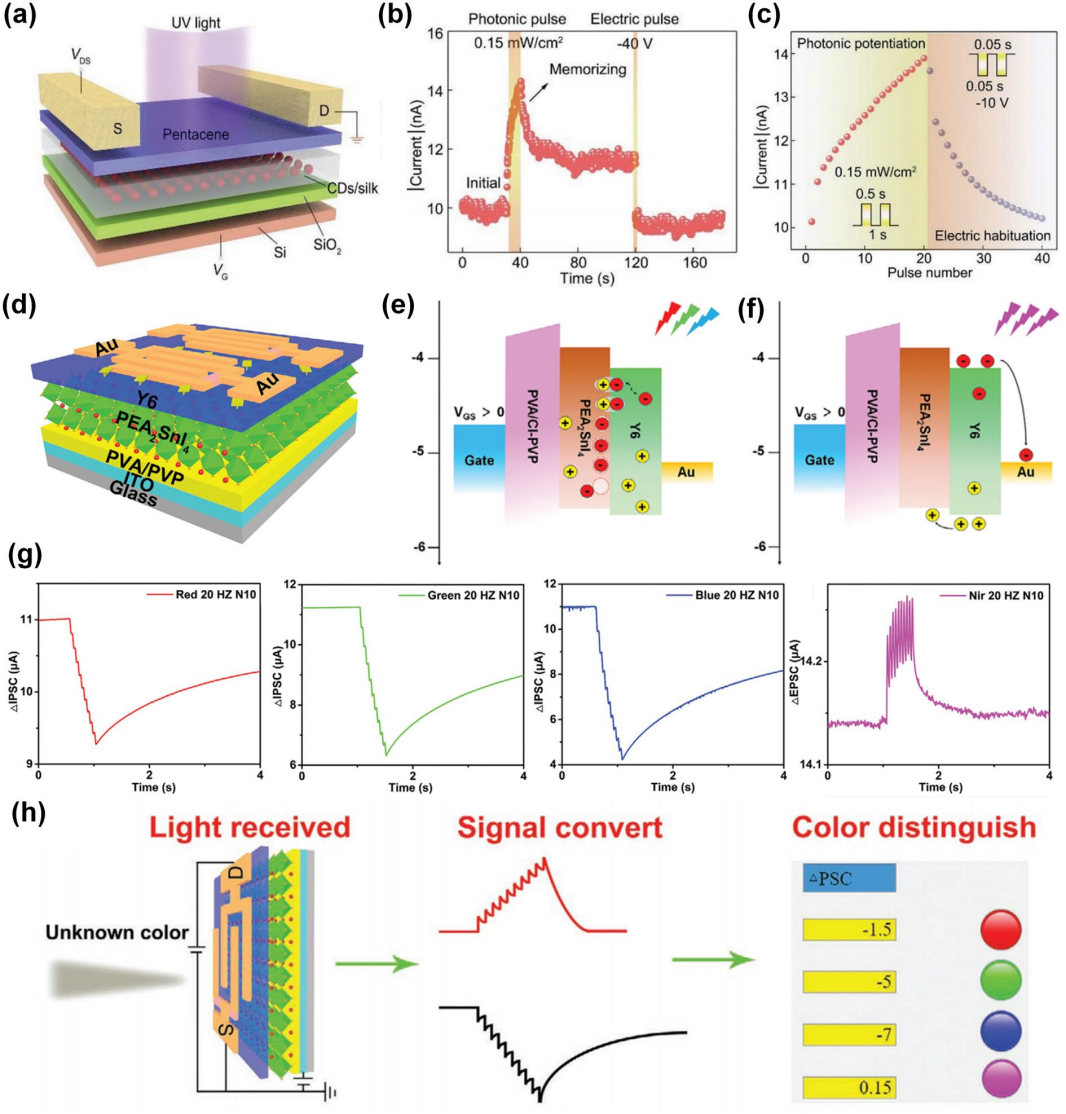
Dynamic plasticity realized in synaptic transistors based on hybrid channel. **a** Schematic of the CDs/silk-based optoelectronic flash memory. **b** Readout current of the such device under the stimulation of input optical and electrical pulses. **c** Photonic potentiation and electric depression triggered series of optical pulses and negative gate pulses [113]. Reproduced with Permission. Copyright 2019, WILEY–VCH Verlag GmbH & Co. KGaA, Weinheim. **d** Device structure of synaptic transistor based on PEA_2_SnI_4_/Y6 hybrid channel. Work mechanism of as-fabricated synaptic transistor under **e** visible light stimuli and **f** NIR light stimuli at *V*_*GS*_ = 40 V, respectively. **g** IPSC triggered by ten visible light spikes at *V*_*GS*_ = 40 V and *V*_*DS*_ = 40 V and the EPSC triggered by NIR light spikes. **h** Simulated process of the synaptic transistor’s recognition of unknown light [37]. Reproduced with Permission. Copyright 2021, WILEY–VCH Verlag GmbH

Comprehensive sophisticated synaptic functions generally rely on the designed functional layers, which was usually composed of hybrid channel layers or insulating dielectric layers in a transistor device. For example, a chitosan electrolyte and a Ta_2_O_5_ high-k dielectric thin film were stacked in a bottom-gate dielectric as an electric double layer (EDL) to trigger the ionic excitatory post-synaptic current [112]. In this work, Ta_2_O_5_ high-k dielectric thin film passivated the organic chitosan electrolyte and improved the gate effect, while the mobile ions in the chitosan electrolyte driven by the gate electric field induced the change of channel conductance to emulate the synaptic plasticity. Lv et al. proposed a carbon dots/silk protein (CDs/silk) hybrid functional layer for gate dielectrics to achieve a light-tunable charge trapping (Fig. 8a) [113]. In this work, the non-volatile change of channel conductance triggered by pre-synaptic light stimuli was attributed to the photo-gating effect induced by the trapped photo-generated electrons in hybrid CDs/silk. As shown in Fig. 8b, an excitatory post-synaptic current was achieved upon the photonic pulse and remained at a higher conductance state compared with the initial state, demonstrating a long-term plasticity, which could be reset to the initial state by applying a negative electric pulse. In this way, photonic potentiation and electric habituation could be realized by applying consecutive photonic pulses followed by electric pulses (Fig. 8c), which represented the synaptic weight updating rules and affected the synaptic transmission efficiency.

Besides the hybrid dielectric layer, a hybrid channel is another choice for designing multifunctional transistors with the assistance of energy band engineering [114–117]. Based on such an idea, Huang et al. reported an ambipolar synaptic transistor based on a 2D perovskite/organic heterojunction (PEA_2_SnI_4_/Y6) hybrid channel, which displayed a dual-mode learning process under light stimulation (Fig. 8d) [37]. When a positive voltage was applied to the bottom-gate, the electron–hole pairs under the visible light illumination could be generated in both 2D perovskite and Y6 layers. In this process, the photo-generated electrons tended to be captured by the Sn vacancies in the 2D perovskite film, while induced more holes had the possibility to be recombined with parts of the electrons in channel, resulting in a decreased channel conductance, namely inhibitory post-synaptic current (Fig. 8e). However, when the device was irradiated with near-infrared, electron–hole pairs could be only generated in Y6 layer, where the photo-generated holes would flow from Y6 into 2D perovskite driven by the difference of band structure, resulting in the accumulation of electrons and an enhanced channel conductance, representing the excitatory post-synaptic current (Fig. 8f). This wave-length dependent conversion between inhibitory and excitatory post-synaptic current (IPSC and EPSC) has been well demonstrated under the visible and infrared pre-synaptic light spikes, respectively (Fig. 8g). Such idea was also utilized to construct a biomimetic eye visual system for color recognition by distinguishing the type of excitement and the magnitude of the post-synaptic current value (Fig. 8h).

Overall, plasticity modulation techniques realized by device structure design are conducive to manipulating the expression of plasticity more flexibly through programmable operations compared with chemical techniques. The proper structure design for synaptic devices enables them to achieve diverse functions like the biological synapses, which is the foundation for building high-performance neural networks at the hardware level. However, complex structural design will make the preparation of single devices and large-scale neural networks difficult. Therefore, optimizing device structure design while enhancing plasticity modulation capability is an important direction for the development of future neuromorphic devices.

## External Physical Modulation toward Neuromorphic Sensing

**Fig. 9:**
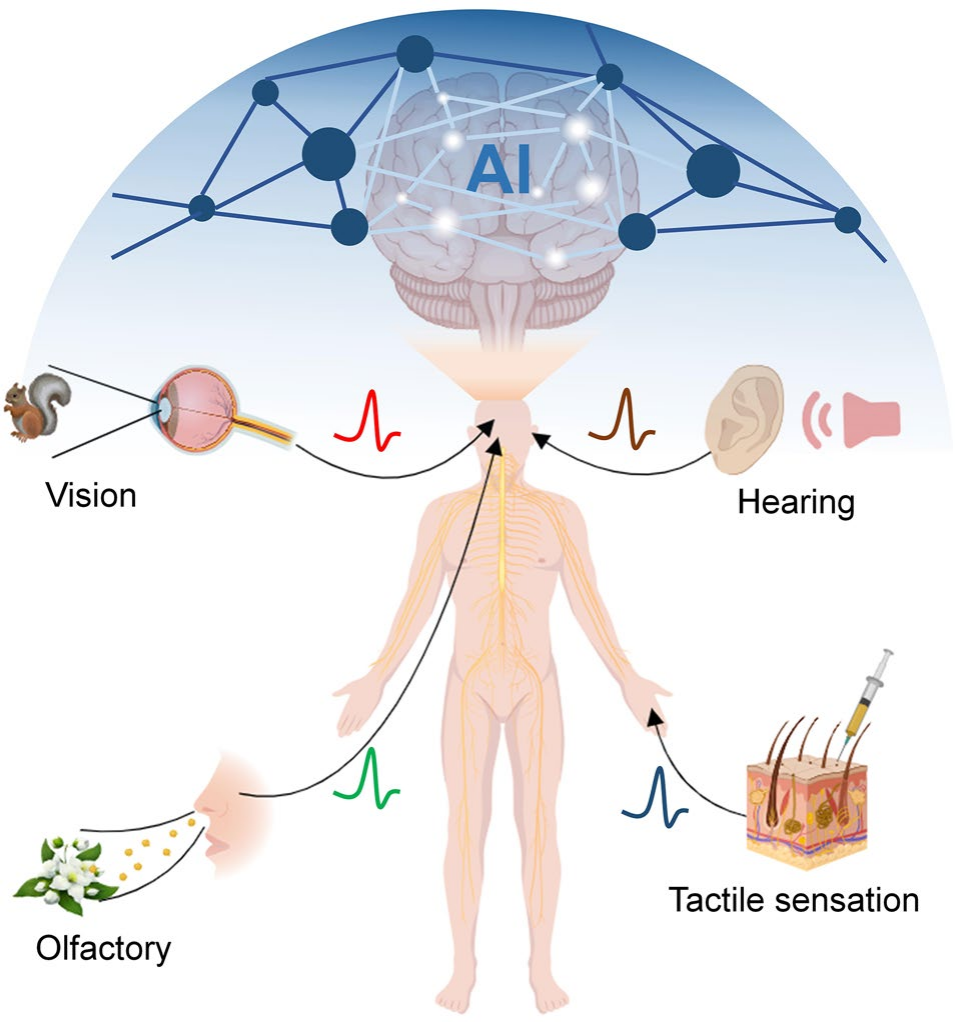
Illustration of perception of external signals by organisms through sensory organs for bio-inspired AI applications

In the human body, a neural system composed of large numbers of synapses is responsible for information processing, learning, cognition, and memory under the modulation of diverse neural activities. Another process is also of great significance for humans and other living creatures, where external stimuli such as light, temperature or strain et al. could be received by the receptors in the sensory organs. The external physical signals are encoded as neural spikes and processed by neural systems with the functions of adaptation, filtering, amplification, and memory, and then transmitted to the cerebral cortex for achieving perception, classification, and identification [118–120]. It also inspires the basic principle to realize the artificial intelligence functions, including vision, tactile sensation, auditory and olfactory as illustrated in Fig. 9. Recent great efforts have been devoted to developing neuromorphic sensing systems based on artificial synaptic devices or neuron devices due to their unique advantages over conventional sensing systems, such as low energy consumption, good adaptability to changing external environments, excellent robustness to noise, variations, and uncertainties in the input data, and real-time processing of data with less data redundancy [121–123]. However, the conversion from physical signals to neural spikes in a biological way is extremely complex, and may involve various neural modulation processes. Because most of the signals in living organisms come from a vision and tactile sensation, we review artificial neural systems with the ability to realize the conversion between external physical signals including light, strain and temperature and neural spikes by plasticity modulation techniques and clarify their physical mechanisms.

### Optogenetics-Inspired Neuromorphic Visions

Photoelectric synapses have attracted much attention due to the wider bandwidth, lower crosstalk, and better scalability of light stimuli instead of electric ones [124–126]. In most reported works, light spikes are applied as pre-synaptic stimuli and the non-volatile change of channel conductance induced by the trapping/de-trapping process of photo-generated carriers represents the synaptic plasticity [127–129]. Such light-stimulated synaptic devices emulated the signal transmission from light spikes to electric spikes in a synapse-like way, which promoted fast neuromorphic computing with low crosstalk and high bandwidth. Light signals can not only serve as input signals but also as control signals, which is consistent with the biological behaviors described by optogenetics [130]. In biological systems, optogenetics highlights the role of light in controlling the generation of proteins or neurotransmitters, which further activate the ion channels and modify the plasticity, resulting in the generation of vision. In this section, we mainly discussed the optogenetics-inspired plasticity modulation techniques, where light worked as a controller to determine the expression of synaptic plasticity for potential applications in neuromorphic visions. Neuromorphic visual systems need to develop new materials and devices to achieve a different mechanism of light-matter interaction from traditional optoelectronic devices [131].

**Fig. 10:**
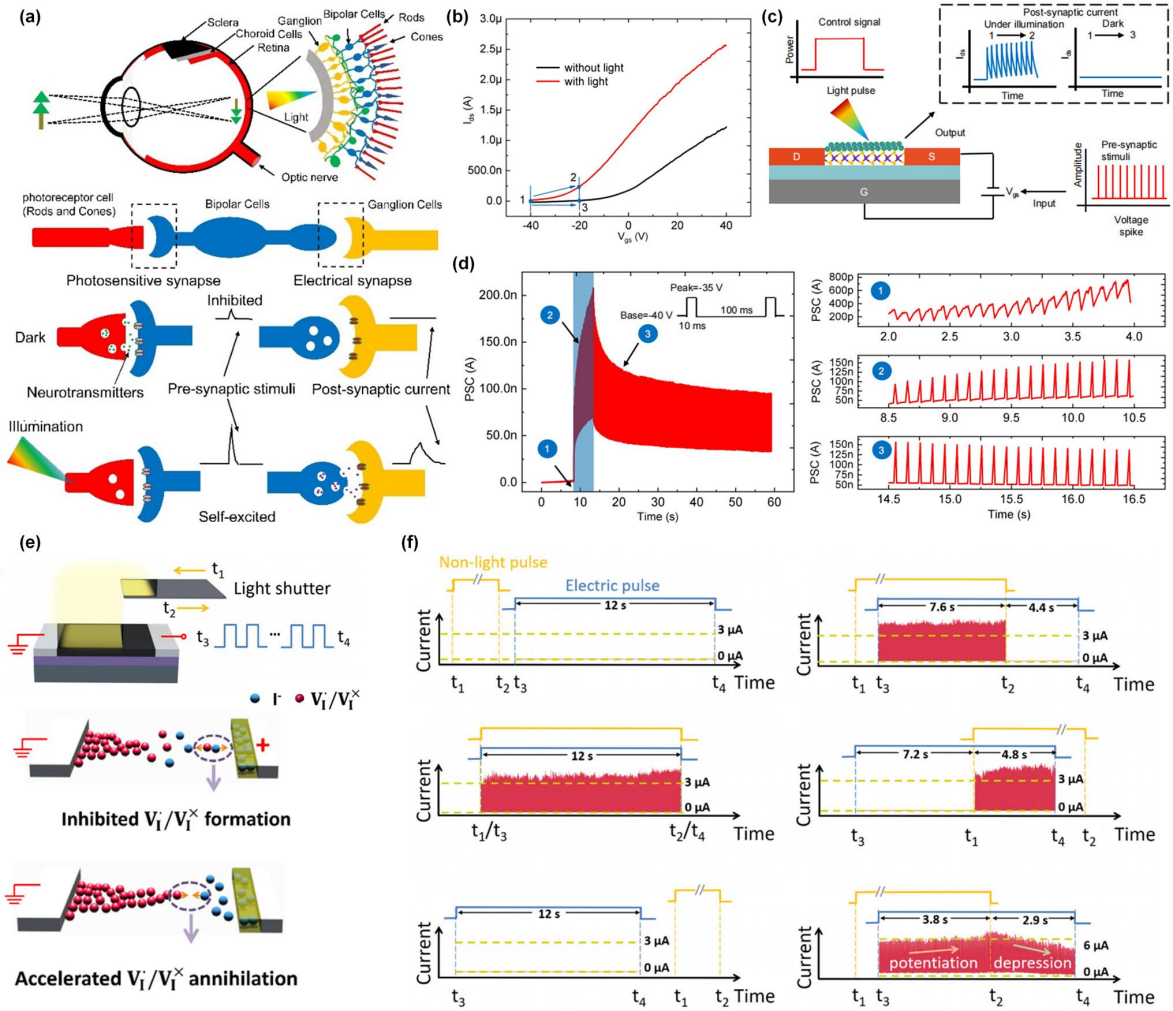
Light-modulated plasticity in artificial synaptic devices. **a** Schematic of the synaptic signal transmission of a biological retina with a three-layer cell structure. **b** Transfer curves of MoS_2_/QDs MD vdW heterostructure-based synaptic transistor in dark and under the illumination. **c** Illustration of light-modulated response of conducting channel to the bottom-gate voltage spikes. **d** Time evolution of PSC triggered by gate voltage spikes under the modulation of illumination [39]. Reproduced with Permission. Copyright 2021, Wiley–VCH GmbH. **e** Schematic of the MAPbI_3_ memristor measured under light illumination and the mechanism for the light-controlled formation and inhabitation of vacancies. **f** Coincidence detection of electrical and light stimulations, exhibiting the light-controlled response to electrical stimulations [132]. Reproduced with Permission. Copyright 2018, American Chemical Society

Figure 10a shows a typical retina with a three-layer structure, namely photoreceptor cell (Rods and Cones), bipolar cell and Ganglion cell [39]. In the dark, the neurotransmitters are realized into the photosensitive synapse and the bipolar cells will be inhibited, resulting in no signal transmission through electrical synapse between bipolar cells and ganglion cells, indicating no neural actions are generated from ganglion cells. However, under illumination, the reflected light from external objects will restrict the release of neurotransmitters from photoreceptor cells and the bipolar cells can be self-excited to realize the neurotransmitters into the electrical synapse, triggering the neural actions in the ganglion cells, which are further transmitted along neural systems into the cerebral cortex to form vision. It can be inferred that the generation of vision is primarily determined by the light-controlled synaptic plasticity. In short, no post-synaptic current is triggered in the dark but continuous post-synaptic current can be obtained under illumination.

Inspired by this idea, a mixed-dimensional (MD) heterostructure based on MoS_2_/QDs has been proposed to emulate the optically modulated synaptic plasticity in an optogenetic way (Fig. 10b, c) [39]. From the transfer curves, the shift of threshold voltage indicated the photo-gating effect dominated the response of such devices to the illumination, which demonstrated that light can control the switching characteristics of FETs. A detailed working principle is illustrated in Fig. 10c, where when a specific train of bottom voltage spikes was applied to the gate terminal in the dark, the channel was pinched off, resulting in no post-synaptic current. Under illumination, when the same voltage spikes were applied, the post-synaptic current could be obtained due to the ON states of the channel caused by light. This idea has been experimentally demonstrated as shown in Fig. 9d, where three regions represent the silent mode in the dark, the working mode under illumination, and the recovery mode after illumination. Besides, similar optically modulated synaptic behaviors were achieved in a perovskite-based memristor, where the light controlled the generation and annihilation of iodine vacancy (Fig. 10e) [132]. From Fig. 10f, the post-synaptic current could be only triggered in the dark because the illumination could inhibit the generation of iodine vacancy, resulting in an unchanged high resistance state.

These works reveal the neuromorphic sensing mechanism of simulating the retina of biomimetic organisms using the photo-gating effect (PGE) at the device level, which paves a new way to develop artificial visual systems. Recently, the PGE has been widely reported in hybrid structures, where layer A is responsible for trapping one type of photo-generated carriers while layer B provides the channel for the transport of another type of photo-generated carriers [133, 134]. In this way, the trapped photo-generated carriers will modulate the potential energy of the channel layer/dielectric interface, resulting in the shift of threshold voltage due to the light-induced additional electric gating effect. Interestingly, the PGE can not only enhance the photoelectric response but also control the switching characteristics of the channel like a transistor gate, providing a theoretical basis for the development of retina-inspired neuromorphic sensors.

### Strain

The tactile perception system of living creatures composed of stimuli receptors and the afferent nervous system can efficiently convert mechanical stimuli from surrounding environments into physiological responses that could be further delivered into the brain to form the sensory feedback [135–137]. And with this, great efforts have been devoted to developing an artificial tactile perception system that can be embedded into prosthetics and artificial skin to restore the tactile sensation of people with disabilities [138–140]. Besides, with the rapid development of artificial intelligence (AI) and the Internet of Things (IoT), wearable electronics integrating tactile sensors and information processors provide intelligent human–computer interaction solutions to achieve external information perception and human biological information acquisition [141]. Recently, high-performance tactile perception systems with the integration of sensitive elements and spike-based synaptic devices have been proposed to emulate the neuromorphic functions [142, 143]. Here, two featured works were presented to showcase completely different modulation mechanisms.

**Fig. 11:**
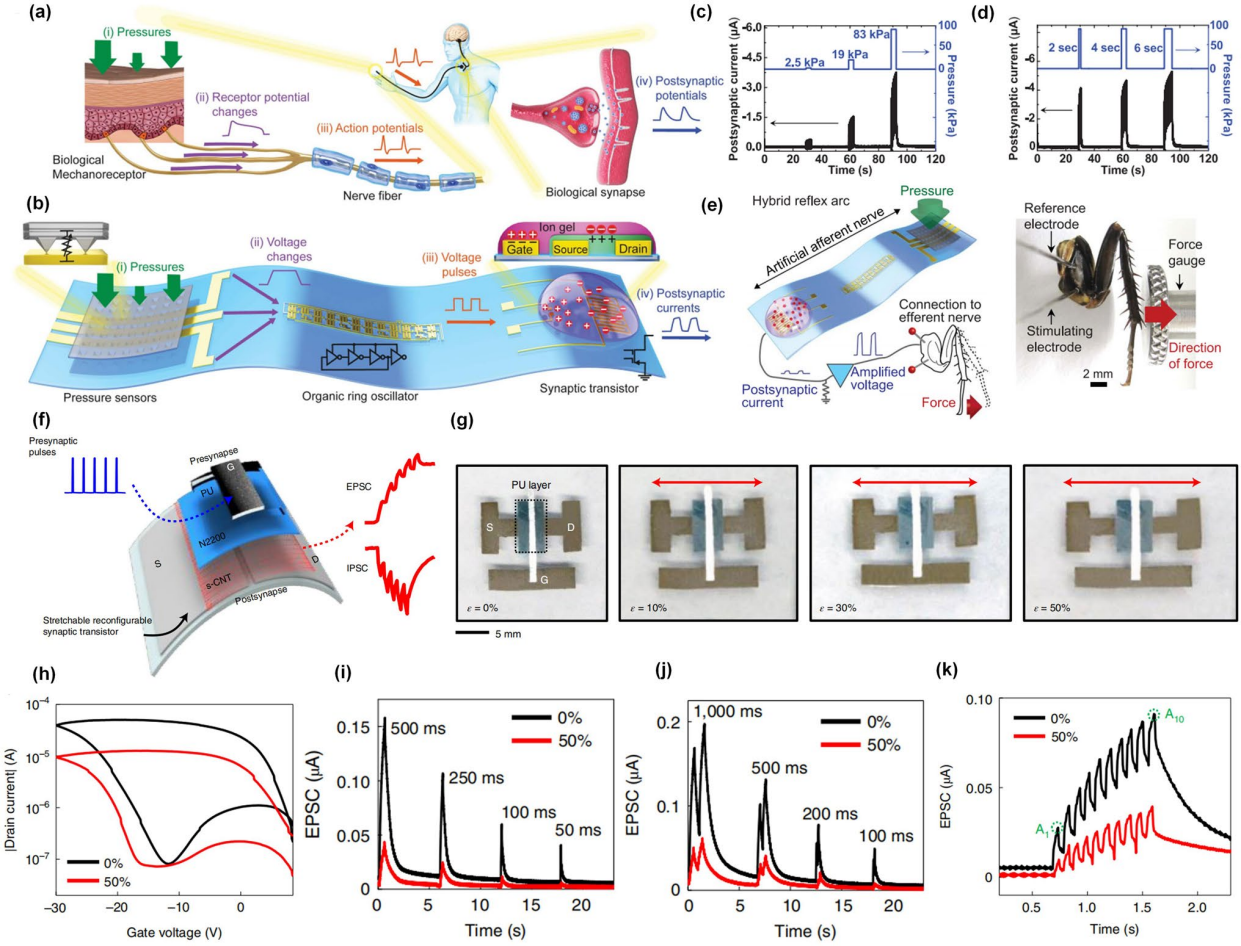
Pressure or strain modulated plasticity in artificial synaptic devices. **a** A biological afferent nerve that is stimulated by pressure. **b** An artificial afferent nerve made of pressure sensors, an organic ring oscillator, and a synaptic transistor. The post-synaptic current under the stimulation of the pressure signals **c** with different strengths and **d** duration, respectively. **e** Hybrid reflex arc made of an artificial afferent nerve and a biological efferent nerve [40]. Reproduced with Permission. Copyright 2018, the AAAS. **f** Schematic of a stretchable reconfigurable synaptic transistor. **g** Optical images of the stretchable reconfigurable synaptic transistor before and after uniaxial stretching by 10%, 30% and 50% along the channel length direction. **h** Transfer curves of such synaptic transistor before and under 50% strain. **i** The EPSC without strain and with 50% strain. **j** EPSC results triggered by two successive pulses without strain and with 50% strain. **k** EPSC results of the device without strain and with 50% strain on the application of ten successive presynaptic pulses [146]. Reproduced with Permission. Copyright 2022, The Author(s), under exclusive licence to Springer Nature Limited

First, inspired by the neuromorphic sensing functions of skins, artificial tactile perception systems are responsible for the converting and encoding of pressure signals into neural spikes. Kim et al. proposed a strategy to fabricate artificial afferent nerves (Fig. 11a) [40], where pressure sensors preliminarily completed the collection of pressure signals and converted them into electrical signals that were further chopped into action potentials with controllable frequency by ring oscillators (Fig. 11b). Finally, the actions potentials were applied to a synaptic transistor as pre-synaptic stimuli to trigger the post-synaptic current. Interestingly, the post-synaptic currents can be directly modulated by the duration and strength of pressure signals (Fig. 11c, d), which help the brain to accurately perceive external pressure information and make appropriate judgments or actions. In this work, the authors successfully built a hybrid reflex arc that could deliver biomimetic post-synaptic signals into the biological efferent nerves in a detached cockroach leg, causing activity of the tibial extensor muscles (Fig. 11e). This work provided a well-demonstrated illustration how to transmit pressure signals into the nervous system of an organism by artificial afferent nerves.

Second, another idea about bridging the strain signals and neural actions was demonstrated in sketchable neuromorphic devices that could exhibit various synaptic functions under the modulation of strains [135, 144, 145]. Different from the afferent nerve shown in Fig. 10a, these works directly applied the strain onto the synaptic devices to control the expression of plasticity. As shown in Fig. 10f, an elastic and reconfigurable synaptic transistor has been fabricated based on a stretchable bilayer semiconductor, which exhibited excellent mechanical flexibility and deformability with strain ranging from 0 to 50% (Fig. 11g) [146]. The strain modulation on the electrical performance and synaptic functions has been also well investigated (Fig. 11h–k), indicating that such devices could retain synaptic functions even when stretched by 50%. Besides, the strength of applied strain could directly affect the synaptic weight change under the same pre-synaptic stimuli, demonstrating the coupling effect between strain and plasticity.

Overall, strain engineering provides the opportunity to modulate the lattice structure of active materials in the stretched or compressed states, which directly changes the electrical band structure such as the magnitude and type of bandgaps, known as the piezoresistive effect [147]. In this way, the resistance or conductance of active materials may be modulated by the applied strain, which contributes to the dynamic plasticity of neuromorphic devices due to the coupling between strain end electrical performance. Therefore, it is a feasible strategy to develop artificial electronic skin by integrating the strain-modulated synaptic devices with information processors.

### Temperature

Temperature is another major factor that significantly affects the physiological and mental activities of living creatures because their life activities need to be carried out within a suitable temperature range [148–150]. At the cellular level of organisms, the influence of temperature is more extensive, which involves enzymatic activity, chemical synthesis of neurotransmitters, switching of ion channels on cell membranes and synaptic action transmission, further modulating the expression of synaptic plasticity [151]. For example, when the temperature drops below the appropriate value, the release of neurotransmitters would be slowed down, resulting in weakened synaptic connections and inefficient information transmission [152]. However, if the temperature exceeds the acceptable threshold value, the structures of the cell would be inactivated or directly destroyed [153]. Therefore, the synapses of organisms need to maintain their function within a certain temperature range, while considering the sensitive perception of external temperature changes to adjust life activities, the expression of plasticity needs to be highly sensitive to temperature. Of course, it is of great significance to investigate the temperature-modulated synaptic plasticity at the device level for developing neuromorphic computing and bionic perception from artificial hardware.

**Fig. 12:**
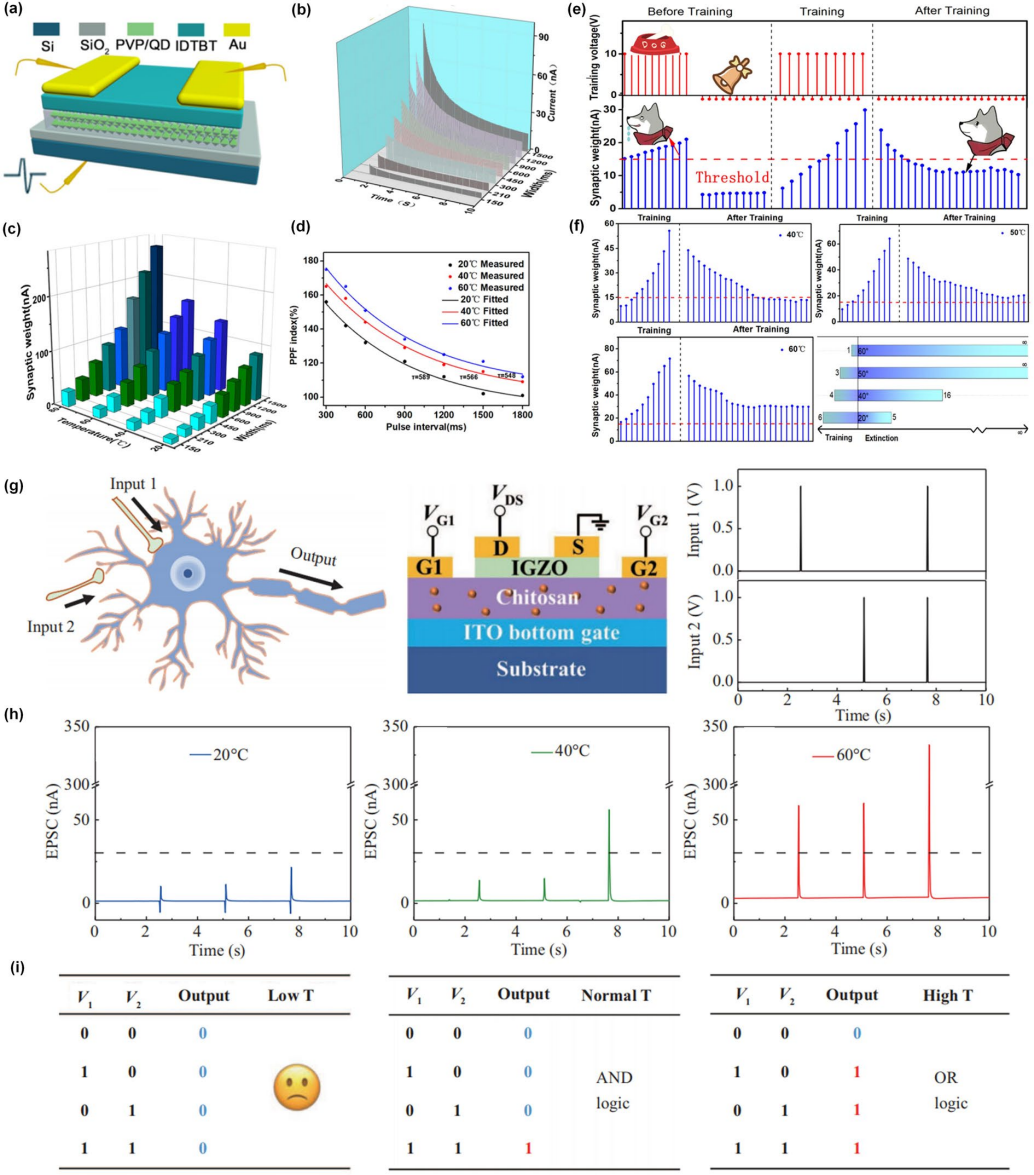
Temperature-modulated synaptic plasticity of artificial synaptic devices. **a** Schematic diagram of the device architecture of the synaptic transistor based on PVP/QDs floating nanogates. **b** Decay of the channel current as a function of time measured after the stimulation. **c** Synaptic weight as a function pulse width measured in different temperatures. **d** PPF index as a function of different temperatures. **e** Stimulation of classical conditioning experiment of Pavlov’s dog at 20 °C. **f** Training and extinction process measured at 40, 50, and 60 °C, respectively, and summary of learning and memory ability of the synaptic transistor at different temperatures [154]. Reproduced with Permission. Copyright 2019, American Chemical Society. **g** A schematic illustration of a neuron with two synaptic inputs and one output and the schematic diagram of a multiterminal in-plane lateral coupled synaptic transistor for logic signal processing. **h** Output current triggered by the presynaptic pulses at 20, 40, and 60 °C, respectively. **i** Truth tables at 20, 40, and 60 °C, respectively [41]. Reproduced with Permission. Copyright 2022, Science China Press and Springer-Verlag GmbH Germany, part of Springer Nature

As mentioned above, neuromorphic electronics should maintain their synaptic functions at an appropriate temperature and their plasticity dynamics could be realized by changing temperature, which means that temperature-sensitive units need to be integrated into such devices. Figure 12a shows a floating-gate organic synaptic transistor with the polyvinylpyrrolidone (PVP)-mixed QDs as floating-gate layer and indacenodithiophene-co-benzothiadiazole (IDTBT) as conducting channel, which exhibited the typical synaptic behaviors triggered by voltage spikes with different width due to the charge trapping effect through floating nanogates (Fig. 12b) [154]. Moreover, from Fig. 12c, d the synaptic weight was greatly enhanced with the temperature increasing from 20 to 80 °C due to the thermally motivated carriers and the decreased ionization activation energy with the increase of temperatures, which induced more free charge carriers [155]. Interestingly, the simulation mechanism of Pavlov’s dog based on the synaptic functions of such devices and the influence of temperature on the training and extinctive process is illustrated in Fig. 12e. From Fig. 12f, it can be inferred that the higher temperature could reduce the times of training process and strengthen the association between bell stimulation and food stimulation, demonstrating the temperature-facilitated modulation of synaptic plasticity. Another example of temperature modulation on synaptic plasticity is illustrated in Fig. 12g, where an indium-gallium-zinc-oxide (IGZO) based electrical-double layer neuromorphic transistor was given two separate gate terminals (*V*_*G1*_ and *V*_*G2*_) and a train of voltage spikes was in sequence applied onto such two pre-synaptic terminals (left panel of Fig. 12g) [41]. The EPSC triggered by the input voltage spike trains under different temperatures is shown in Fig. 11h and the threshold value was set to be 30 nA. From the results, the devices exhibited no response at 20 °C and worked in a mode of “AND logic” at 40 °C, which was further developed into the operation of “OR logic” at 60 °C (Fig. 12i). The underlying mechanism could be attributed to the temperature modulated movement of proton migration in the chitosan dielectric layer. In this way, the temperature could be seen as a modulatory parameter to control the spiking logic switching, which is crucial for synaptic transmission.

The correlation between temperature and synaptic plasticity can be attributed to the thermally motivated carriers and temperature-dependent charge trapping/de-trapping process [155, 156]. With the increasing temperature, more free charge carriers could be induced, which intensifies the response of neuromorphic devices to presynaptic stimuli, thereby leading to enhanced synaptic weight. Besides, the synaptic plasticity of many reported neuromorphic devices was achieved by the charge trapping/de-trapping process, which caused the origin of STP and LTP. The retention time of STP and LTP is related to the temperature, which can be further understood by the relationship between the relaxation time (τ) and activation energy (*E*) with the temperature (*T*) as shown below [154]:

$$A + \ln \left( {\frac{1}{\tau \left( T \right)}} \right) = \frac{E}{kT}$$

where *E* stands for the activation energy,* k* is the Boltzmann constant, and *A* acts as a stochastic parameter. The higher temperature will lower the activation energy and accelerate the process of charge de-trapping, resulting in the transition from LTP to STP. In this way, temperature can be used to control the synaptic weight and memory behaviors of neuromorphic devices, which can conversely reflect temperature changes.

From this section, we reviewed the modulation of external physical signals such as light, strain and temperature on the synaptic plasticity, which plays an important role in controlling the synaptic signal transmission and biomimetic perception function. The interaction between external physical signals and synaptic plasticity has been clarified to illustrate the underlying physical mechanisms for plasticity modulation techniques, which lays the foundation for artificial intelligence perception systems. Besides, artificial olfactory synaptic devices have been proposed to investigate the synaptic functions when exposed to target gases, which provided a new way to realize gas recognition based on the specific synaptic behaviors induced by gas response [157, 158]. Chen et al. reported a novel artificial neuron-like gas sensor based on CuS QDs/Bi_2_S_3_ nanosheets (NSs), where CuS QDs provided adsorption sites for target gas molecules and Bi_2_S_3_ NSs worked as fast charge transport channels [159]. In this work, as-fabricated artificial neuron-like sensors can be stimulated by gas in a way of biological olfactory perception. The core idea of designing such devices is how to establish the connection between physical signal sensitivity characteristics and neural behavior and achieve the transmission and perception of synaptic signals through this method.

## Conclusions and Perspectives

In neuromorphic devices, the purpose of plasticity modulation is to use technological means to simulate the complex neural activities of organisms. Therefore, synaptic plasticity modulation is an important pathway for achieving diverse neuromorphic functions, which are especially important for achieving reconfigurable neuromorphic computing and intelligent perception of similar organisms. In principle, the controllable expression of plasticity is achieved through the modification of functional materials, the functionalization of device structures, and the response of substances to external stimulus signals. This makes it possible to manipulate the plasticity expression of neuromorphic devices through chemical methods, device structure design, and physical signal regulation as discussed in this review. However, the existing methods of plasticity modulation are relatively monotonous, and related research is still in the exploratory stage. Therefore, this review provides the following prospects for the future development trends of plasticity modulation techniques.

### Enriching Plasticity Modulation Mechanism

The high intelligence of organisms depends on the complex modulatory mechanisms of the nervous system. However, the structural complexity of artificial neuromorphic devices is far lower than that of the neurons and synapses of organisms. Moreover, artificial neuromorphic devices cannot achieve the modulation level like organisms. In fact, for artificial neuromorphic hardware inspired by the information processing and intelligent perception methods of the nervous system of organisms, its performance can be significantly improved without pursuing the requirements of organisms completely. Currently, the main results of plasticity modulation are still focused on the conversion between STP and LTP, the controllable expression between excitatory and inhibitory plasticity, as well as the improvement of symmetry and nonlinearity for synaptic weight updating. Such limitation on the modulation effectiveness is mainly due to the lack of reliable, flexible, and diverse plasticity modulation mechanisms from the device level. Therefore, enriching the plasticity modulation mechanisms of neuromorphic devices will be conducive to achieving breakthroughs in operating speed, power consumption, and functional diversity of neuromorphic chips.

### Efficient Modulation Techniques for Scaled Neural Networks

It is a consensus that only when neuromorphic devices are used to construct large-scale networks can their advantages in information processing be fully utilized. Although the study of plasticity modulation for individual devices can enrich the modulation mechanism and methods, it is still necessary to consider how to achieve selective modulation and its reliability for large-scale neuromorphic device arrays in practical applications. From this point of view, chemical methods make it easier to achieve large-scale performance modulation by introducing doping during the synthesis stage of functional materials or modifying devices through surface treatment to achieve the synaptic plasticity modulation. However, as mentioned in Sect. 2, the disadvantage of chemical methods is that once doping is completed, the properties become fixed, which is not conducive to the adaptive and self-regulation characteristics of neural networks. Thus, it is a practical and feasible research solution to achieve reconfigurable modulation of neuromorphic chips using voltage bias signals through circuit structure design. For this solution, it is necessary to consider how to reduce the issues of area, power consumption, and process complexity caused by the introduction of additional control circuits.

### Multimodal Collaborative Plasticity Modulation Techniques

**Fig. 13:**
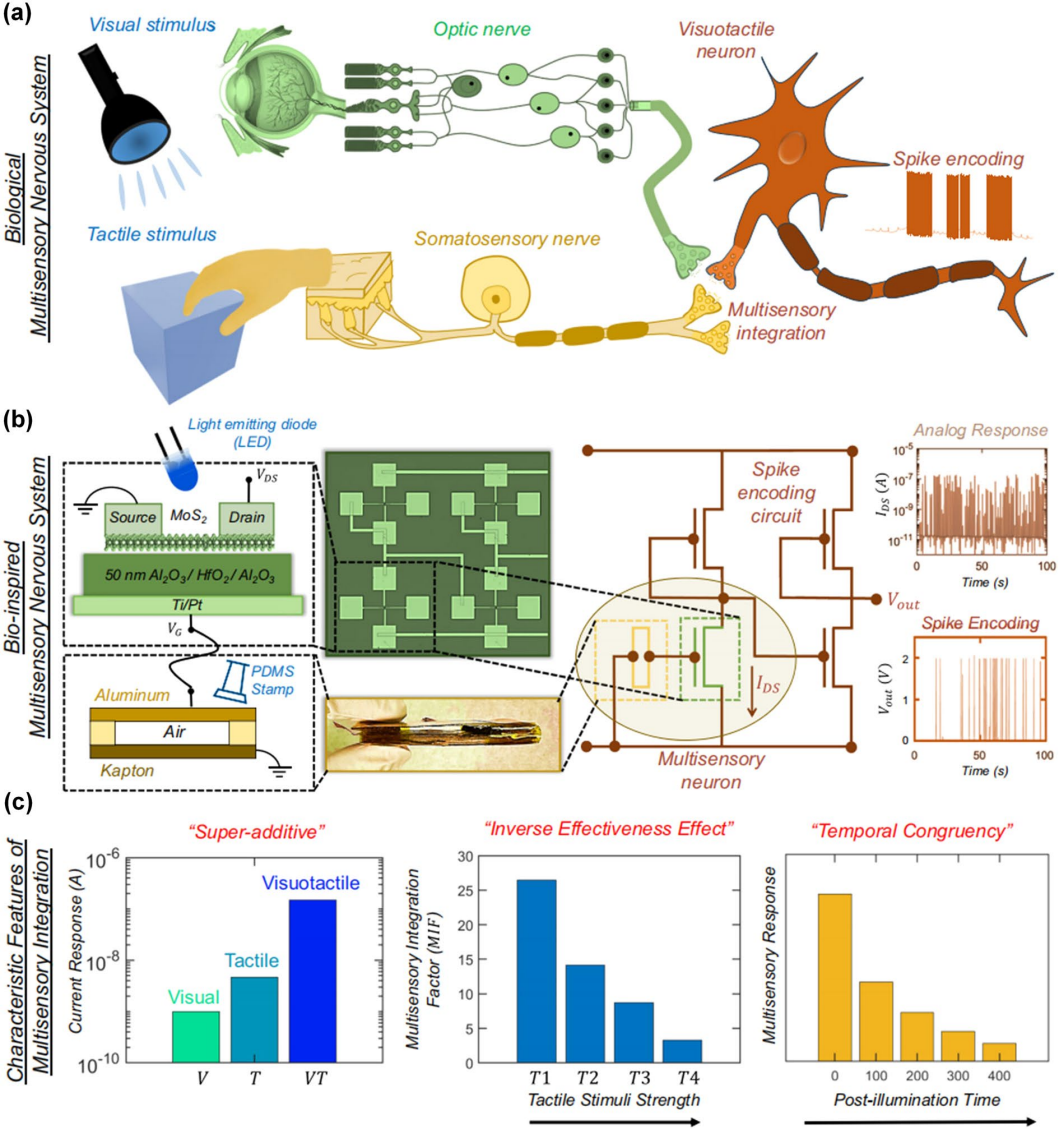
A concept demonstration of light and pressure co-modulated synaptic devices. **a** Schematic representation of multisensory integration of visual and tactile information within the biological nervous system. **b** A bio-inspired visuotactile multisensory neuron (MN) comprising a triboelectric tactile sensor connected to the gate terminal of a monolayer MoS_2_ photomemtransistor along with the associated spike encoding circuit. **c** The three characteristic features of multisensory integration, i.e., super-additive response to cross-modal cues, inverse effective effect, and temporal congruency, are demonstrated by such multisensory devices [160]. Reproduced with Permission. Copyright 2023, Springer Nature Limited

Compared with neuromorphic computing, neuromorphic sensing needs to consider the perceptual integration of multiple physical signals, because organisms never perceive a single signal when receiving external information. They often combine the fusion of multisensory information, such as vision, hearing, and touch, and achieve an understanding of the world through comprehensive processing by the brain. This requires that when designing and developing neuromorphic sensors, consideration should be given to the conversion mechanisms of various physical signals to neural signals and the collaborative processing mechanisms of the signals from different sensory units. Recently, Saptarshi Das et al. designed and fabricated a visuotactile neuron device with the integration of a photosensitive monolayer MoS_2_ memtransistor and a triboelectric tactile sensor [160]. The main idea of this work is illustrated in Fig. 13a, where the photosensitive unit was responsible for the conversion of the visual stimulus into neural signals and the pressure unit converted the tactile stimulus into neural signals, respectively. Finally, the integration of neural signals from two different sensatory units would be completed in a visuotactile neuron. Figure 13b further clarifies how to achieve such integration of multisensory signals by artificial neuromorphic circuits. The tactile stimuli were encoded into voltage spikes by the triboelectric effect, which was further transcribed into source-to-drain output current spikes in the MoS_2_ photomemtransistor by connecting the output of tactile sensors with the gate terminal of MoS_2_ photomemtransistor. Besides, the photo-gating effect induced by the unique light-matter interaction between MoS_2_ and input illumination contributed to the conversion of visual stimuli into electrical responses. A MoS_2_ memtransistor-based encoding circuit was adopted to complete the integration of spike signals. Interestingly, three characteristic features of multisensory integration, i.e., super-additive response to cross-modal cues, inverse effective effect, and temporal congruency, have been successfully simulated, which highlighted the importance of multisignal perception in achieving complex neural functions (Fig. 13c). Therefore, exploring different neuromorphic sensing mechanisms and multisignal fusion processing is an important research direction for the further development of neuromorphic sensors.

In conclusion, current research works on plasticity modulation techniques are still in its initial stage, and reported plasticity modulation techniques cannot meet the needs of high-performance neuromorphic chips. This review summarized the modulation mechanism of chemical techniques, device structure design and external physical signal sensing, which contributed to the performance improvement and functional diversification of neuromorphic devices for enhanced computing and advanced sensing. A comprehensive review of plasticity modulation techniques from the types of modulation, physical mechanisms, and achievements can enable researchers to quickly select corresponding technologies based on their research objectives. This hot topic could provoke a heated discussion on manipulating the performance of neuromorphic devices, which would bridge the artificial neuromorphic electronics and artificial intelligence.
